# Supplementary material for: Identification of a herpes simplex virus 1 gene encoding neurovirulence factor by chemical proteomics
Source: Nat Commun. 2020 Sep 29;11:4894. doi: 10.1038/s41467-020-18718-9 (PMC7524712; doi:10.1038/s41467-020-18718-9)
Supplement: Supplementary file 1 — Supplementary Information [file 41467_2020_18718_MOESM1_ESM.pdf]

**Supplementary information**

# Identification of a Herpes Simplex Virus 1 Gene Encoding Neurovirulence Factor by Chemical Proteomic

Kato and Adachi et al.

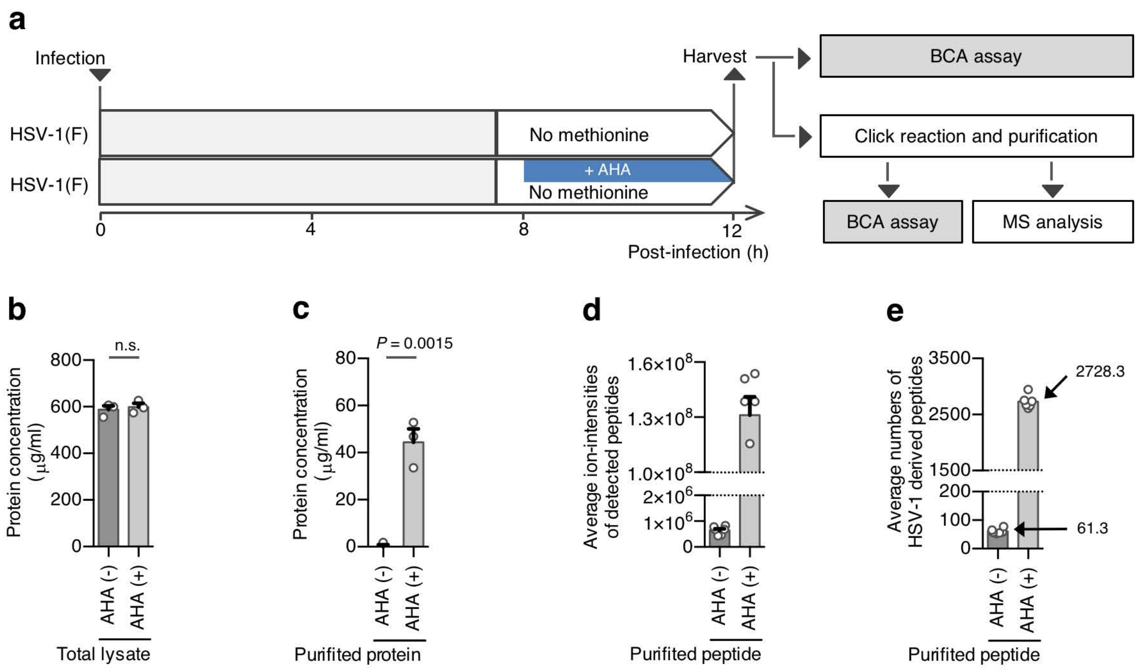


**Supplementary Fig. 1. Purification of newly synthesized proteins by click chemistry. a,** Diagram illustrating the procedure for BONCAT-based MS-analyses including AHA- or mock-labeling experiments, click chemistry, affinity purification and MS-analyses of newly synthesized proteomes (n = 3). In each of the three experiments, MS analyses were performed twice. **b,** Protein concentrations in the lysates of HSV-1 infected cells in the absence or presence of AHA were determined by BSA assay (n = 3). Each value is the mean ± SEM of three independent experiments analyzed by an unpaired two-tailed Student's *t*-test. n.s., not significant. **c to e,** Newly synthesized proteins mock-labeled or labeled with AHA were purified by click chemistry from lysates of wild-type HSV-1(F) infected cells and then subjected to MS-analysis. Peptide concentrations (**c**) were determined by bicinchonic acid (BCA) (n = 3). Each value is the mean ± SEM of three independent experiments. The indicated *P*-values were obtained using two-tailed Welch's *t*-test (**c**). Mean values of ion-intensity of peptides (**d**) and mean numbers of HSV-1 derived peptides (**e**) were determined by MS-analyses. Each value is the mean ± SEM of six MS analyses (**d, e**). Source data are provided as a Source Data file.

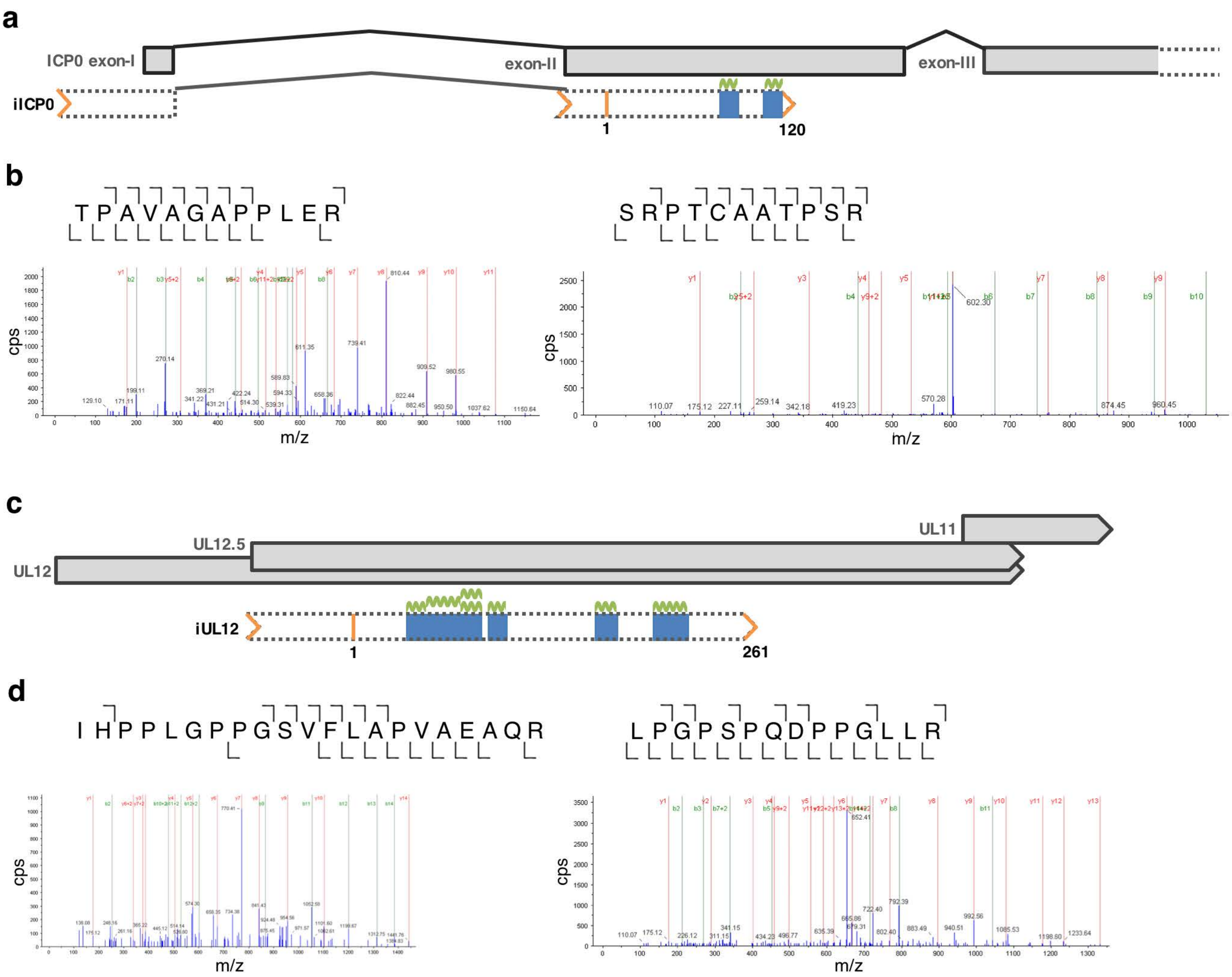

a
ICP0 exon-I
exon-II
exon-III
iICP0
1
120
b
TPAVAGAPPLER
SRPTCAATPSR
cps
m/z
c
UL12.5
UL11
UL12
iUL12
1
261
d
IHPPLGPPGSVFLAPVAEAQR
LPGPSPQDPPGLLR
cps
m/z

e

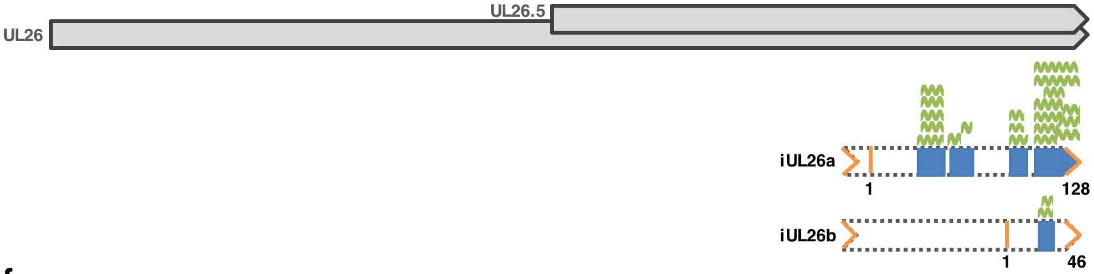
UL26
UL26.5
iUL26a
1
128
iUL26b
1
46

f

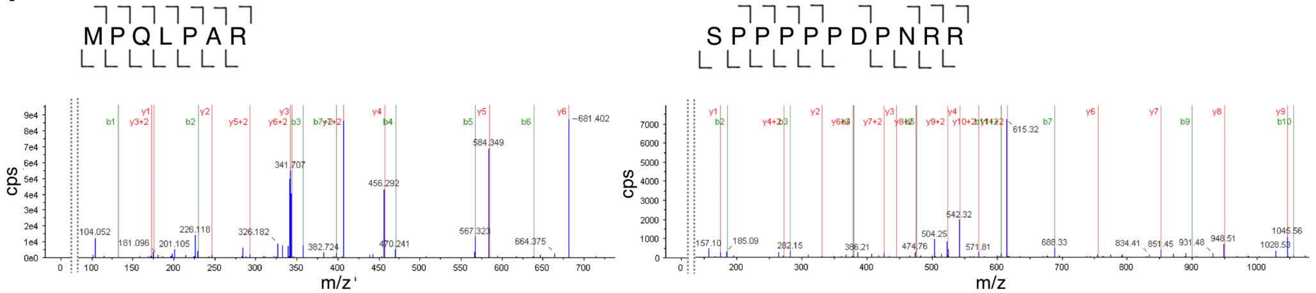
M P Q L P A R
S P P P P P D P N R R
cps
m/z
m/z

g

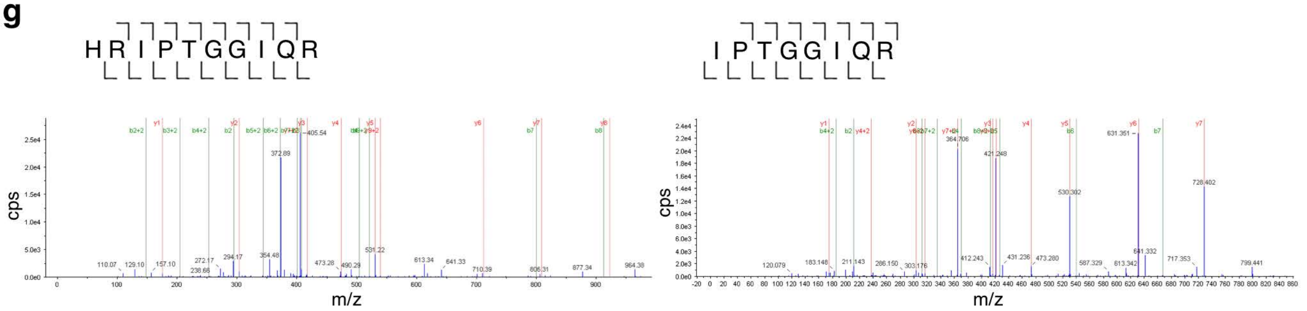
H R I P T G G I Q R
I P T G G I Q R
cps
m/z
cps
m/z

h

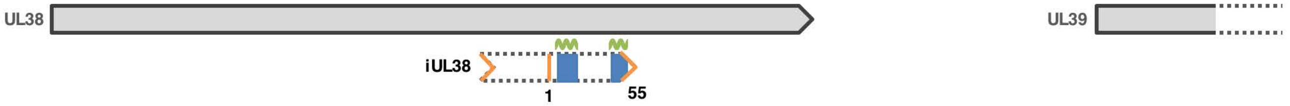
UL38
UL39
iUL38
1
55

i

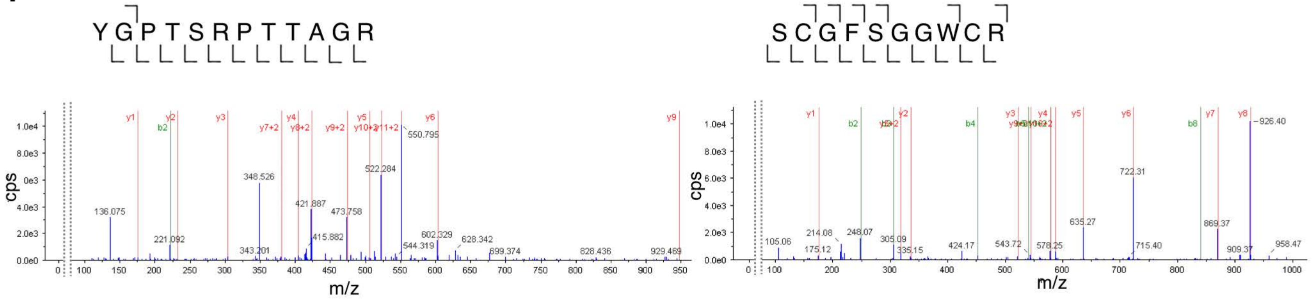
Y G P T S R P T T A G R
S C G F S G G W C R
cps
m/z
cps
m/z

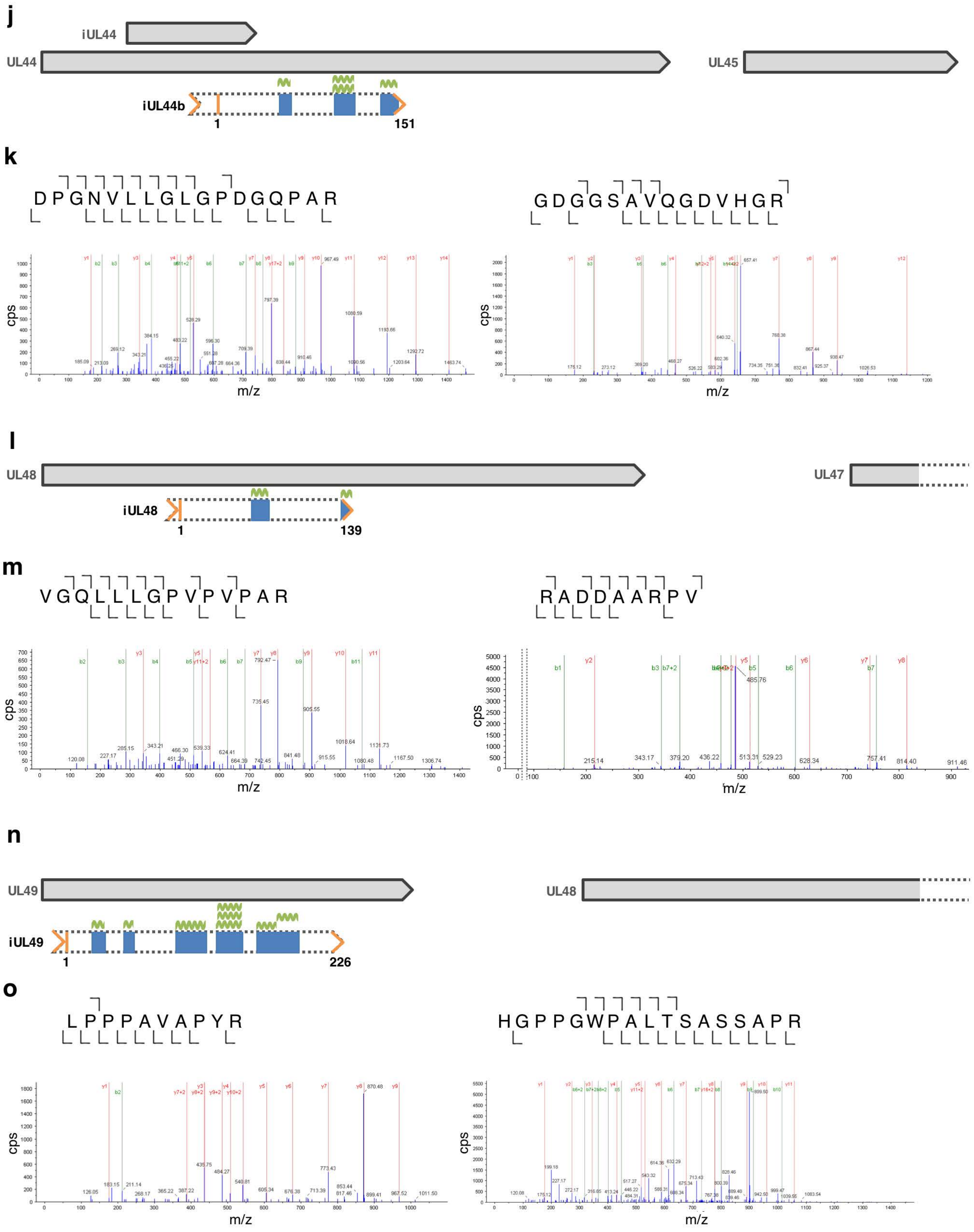

j
iUL44
UL44
UL45
iUL44b
1
151
k
DPGNVLLGLGPDGQPAR
GDGGSAVQGDVHGR
cps
m/z
l
UL48
UL47
iUL48
1
139
m
VGQLLLGPVPVPAR
RADDAARPV
cps
m/z
n
UL49
UL48
iUL49
1
226
o
LPPPAVAPYR
HGPPGWPALTSASSAPR
cps
m/z

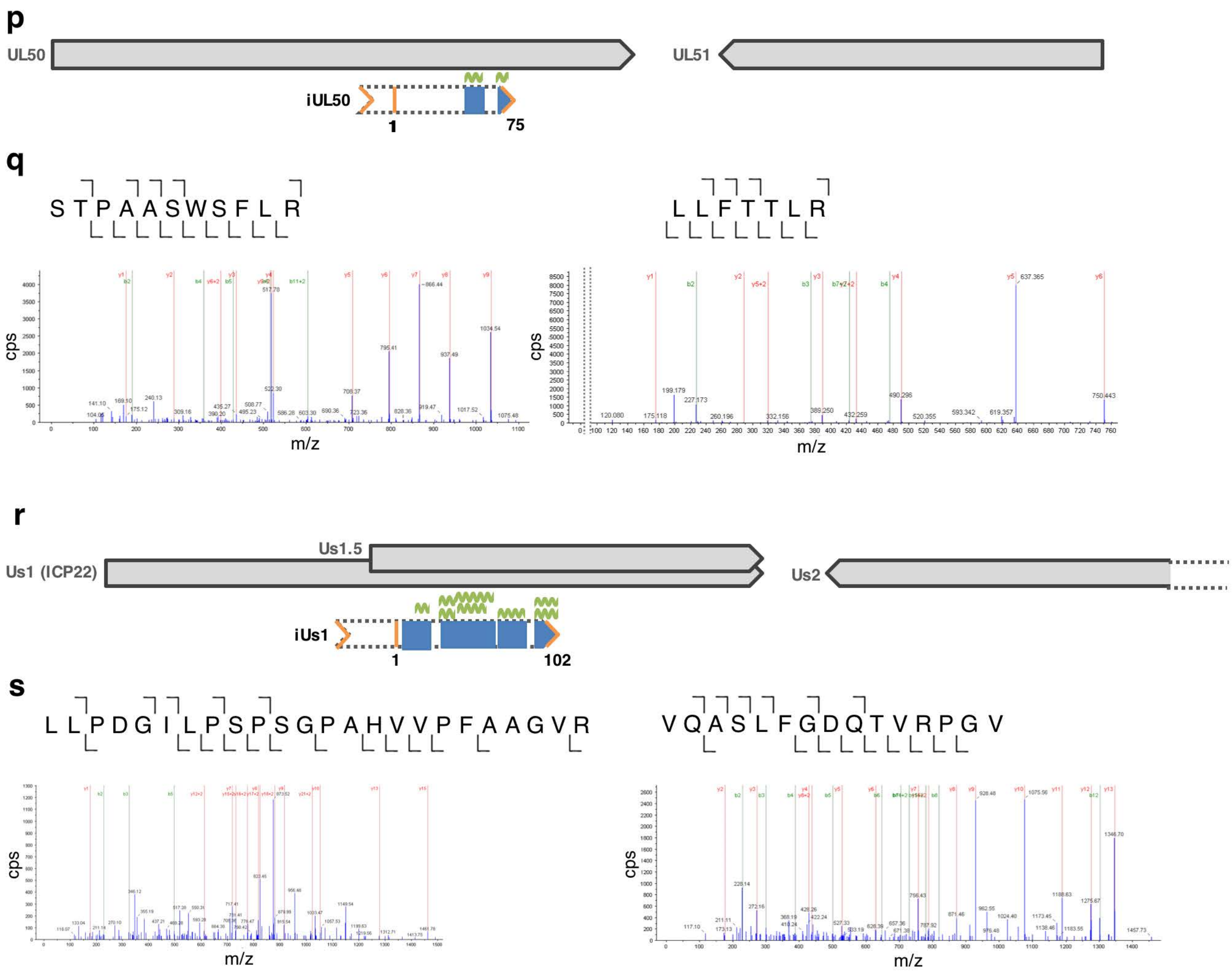


**Supplementary Fig. 2. Identification of previously unidentified HSV-1 CDSs. a, c, e, h, j, l, n, p, r,** Schematic diagram of known HSV-1 CDSs and those identified in this study. The location and orientation of ORFs containing iICP0 (**a**), iUL12 (**c**), iUL26a (**e**), iUL26b (**e**), iUL38 (**h**), iUL44b (**j**), iUL48 (**l**), iUL49 (**n**), iUL50 (**p**) or iUs1 (**r**) are shown by dotted gray lines. Putative translational start codons or stop codons of the identified CDSs are marked by orange vertical bars or close angles, respectively. The MS-detected domains or peptides are illustrated as blue boxes within the CDSs or green wavy lines above the CDSs. Known HSV-1 CDSs are shown in grey close lines. **b, d, f, g, i, k, m, o, q, s,** Representative spectra for the peptides derived from iICP0 (**b**), iUL12 (**d**), iUL26a (**f**), iUL26b (**g**), iUL38 (**i**), iUL44b (**k**), iUL48 (**m**), iUL49 (**o**), iUL50 (**q**) or iUs1 (**s**). cps., count per second.

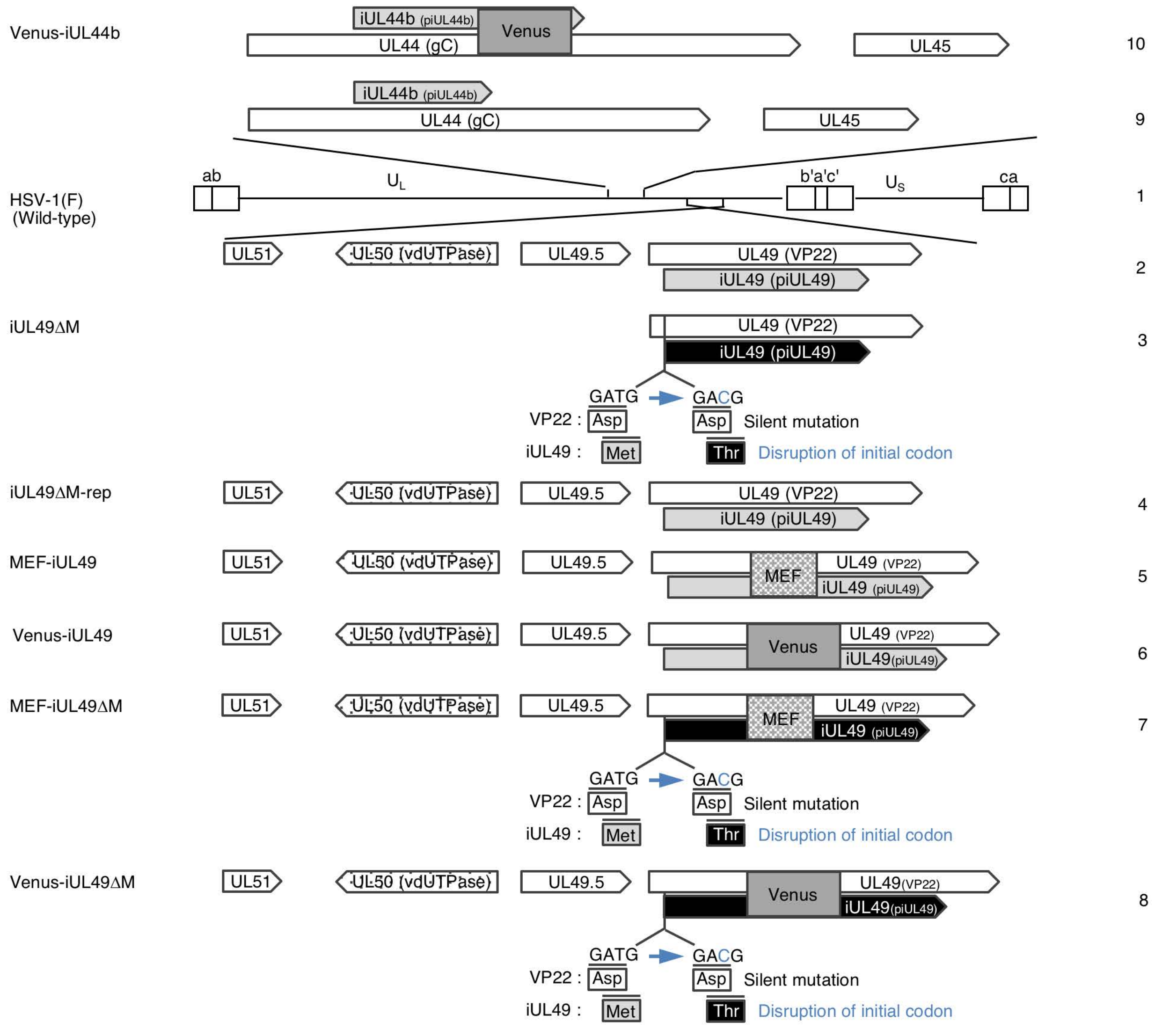


**Supplementary Fig. 3. Genome structure of recombinant viruses-I.** Line 1, the wild-type HSV-1 genome; line 2, the structure of the UL49 (VP22), iUL49 (piUL49), UL49.5, UL50 (vdUTPase) or UL51 CDS; lines 3 or 4, recombinant viruses with a mutation in iUL49; lines 5 or 6, recombinant viruses carrying Venus- or MEF-tagged iUL49 (Venus-iUL49 or MEF-iUL49); lines 7 or 8, recombinant viruses with a mutation in iUL49 in Venus-iUL49 or MEF-iUL49. All substitutions in the iUL49 gene shown in lines 3, 7 and 8 were designed to have no effect on the amino acid sequence of VP22; line 9, the structure of the UL44 (gC), iUL44b (piUL44b) or UL45 CDS; line 10, a recombinant virus carrying Venus-tagged iUL44b (Venus-iUL44b).

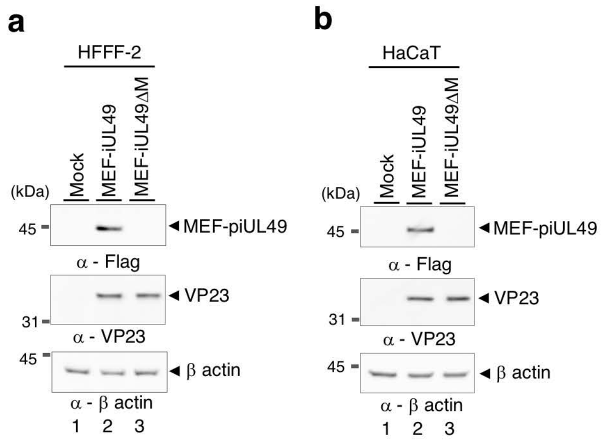


**Supplementary Fig. 4. Accumulation of piUL49 in human fibroblast or keratinocyte cells. a, b** HFFF-2 (**a**) or HaCaT (**b**) cells mock-infected or infected with MEF-iUL49 or MEF-iUL49ΔM for 12 h at an MOI of 10 were analyzed by immunoblotting with antibodies to Flag, VP23 or β actin. Digital images are representative of three independent experiments (**a**, **b**).

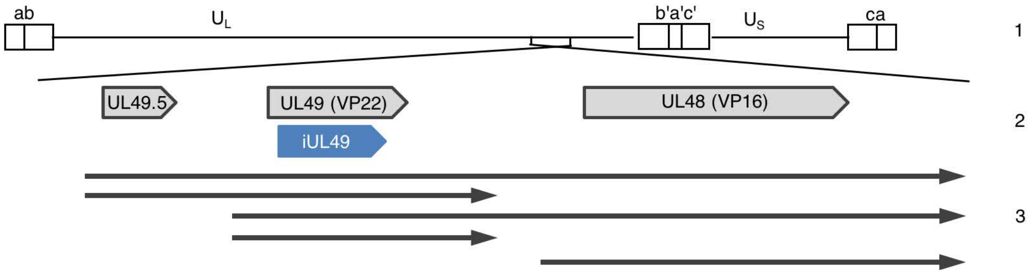


**Supplementary Fig. 5. Reported transcripts around the iUL49 locus.** Line 1, Schematic diagram of the genome structure of wild-type HSV-1(F); line 2, locations of UL49.5, UL49(VP22), iUL49 and UL48(VP16) CDSs; line 3, reported transcripts around the iUL49 locus were indicated by back arrows.

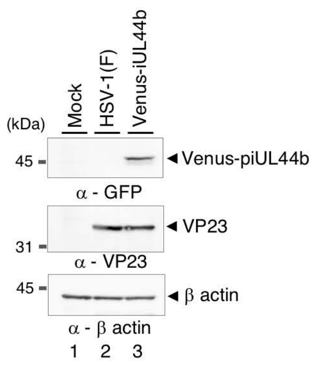


**Supplementary Fig. 6. Expression of piUL44b in HSV-1-infected cells. a,** Vero cells mock-infected or infected with wild-type HSV-1(F) or Venus-iUL44b for 12 h at an MOI of 10 were analyzed by immunoblotting with antibodies to GFP, VP23 or β actin. Digital images are representative of three independent experiments.

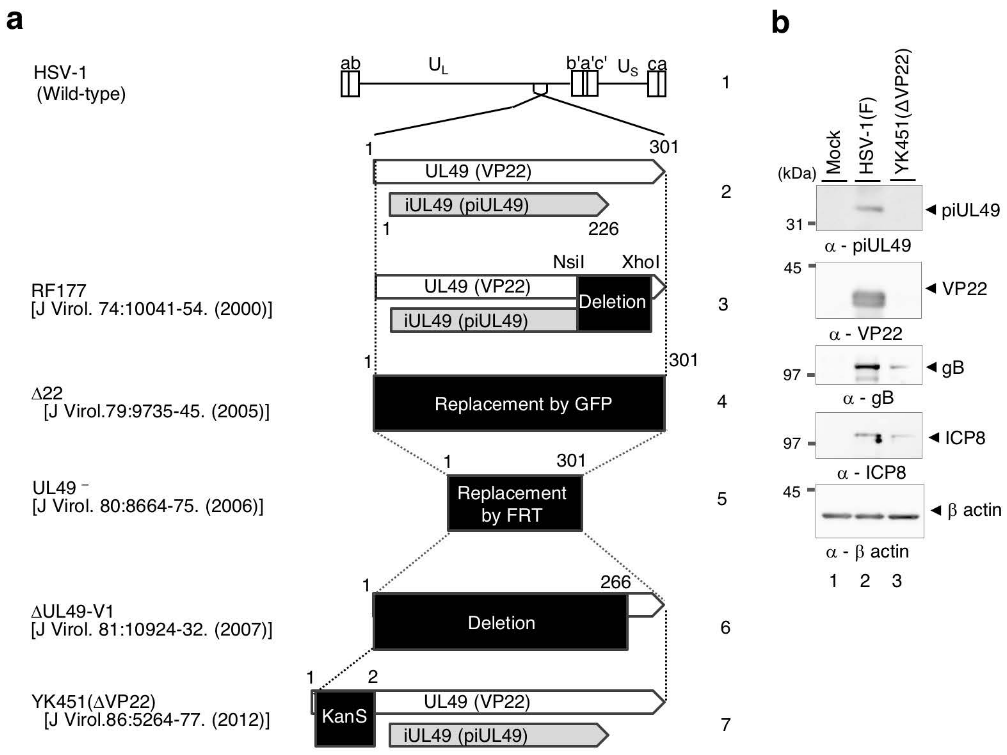


**Supplementary Fig. 7. VP22 null-mutant viruses used in earlier studies. a**, Schematic diagrams of the genome structures of wild-type HSV-1 and UL49 (VP22) null-mutant viruses used in earlier studies. Line 1, wild-type HSV-1 genome; line 2, the structure of the UL49 (VP22) and iUL49 (piUL49) CDSs; lines 3 to 7, recombinant viruses with a mutation in VP22 and/or iUL49. FRT; flippase recognition target. **b,** Vero cells mock-infected or infected with wild-type HSV-1(F) or YK451(ΔVP22) for 12 h at an MOI of 10 were lysed and analyzed by immunoblotting with antibodies to piUL49, VP22, gB, ICP8 or β actin. The VP22-null mutation abolished the expression of piUL49. Digital images are representative of three independent experiments. Source data are provided as a Source Data file.

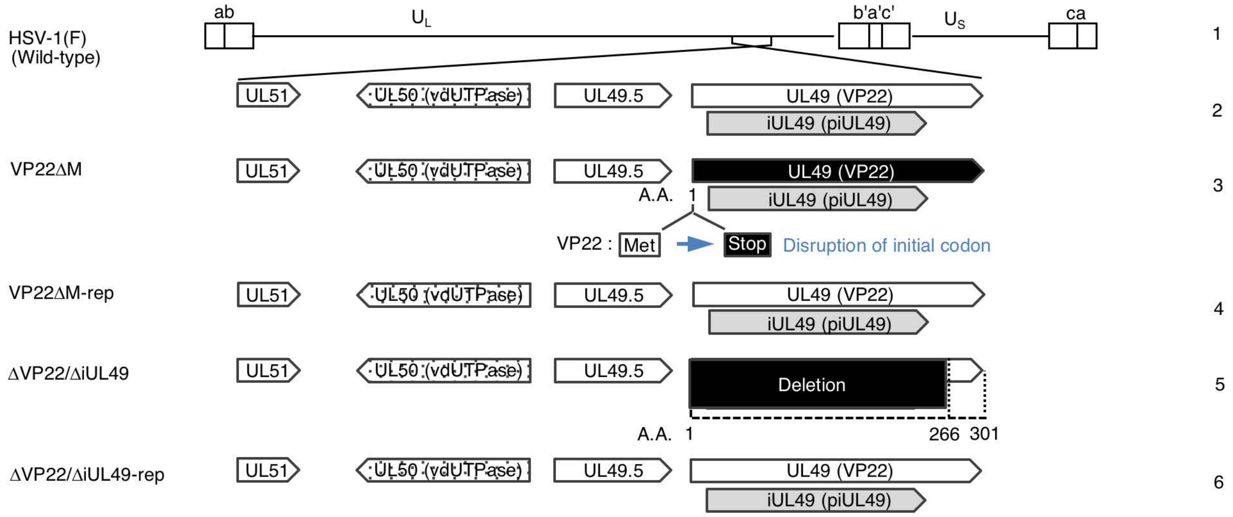


**Supplementary Fig. 8. Genome structure of recombinant viruses-II.** Line 1, the wild-type HSV-1 genome; line 2, the structure of the UL49 (VP22), iUL49 (piUL49), UL49.5, UL50 (vdUTPase) or UL51 CDSs; lines 3 to 6, recombinant viruses with a mutation(s) in iUL49 and/or VP22.

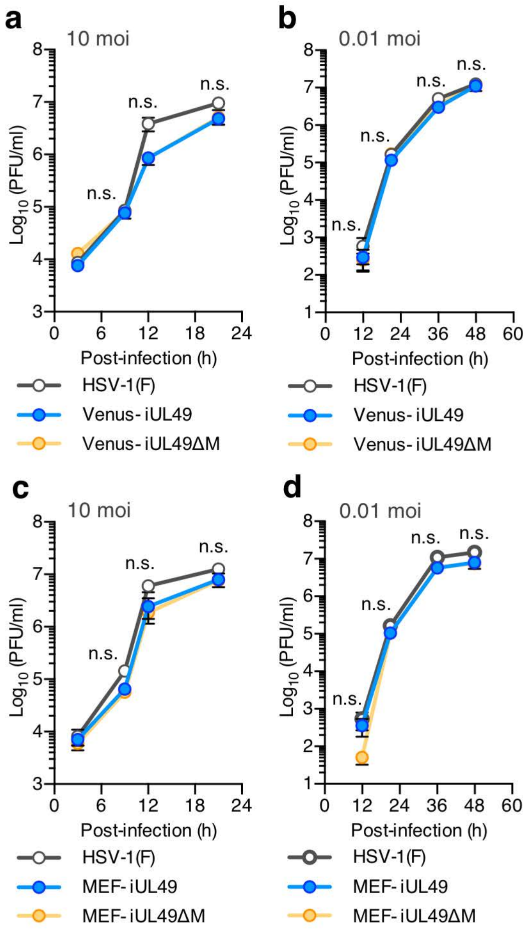


**Supplementary Fig. 9. Effects of taggings of piUL49 on HSV-1 replication. a** to **d,** Vero cells were infected with wild-type HSV-1(F) (**a** to **d**), Venus-iUL49 (**a** and **b**), Venus-iUL49ΔM (**a** and **b**), MEF-iUL49 (**c** and **d**) or MEF-iUL49ΔM (**c** and **d**) at an MOI of 10 (**a** and **c**) or 0.01 (**b** and **d**). Total virus from the cell culture supernatants and infected cells was harvested at the indicated times and assayed. Each value represents the mean ± SEM of three (**a** to **c**) or four (**d**) independent experiments and analyzed by one-way ANOVA followed by Tukey's test. n.s., not significant (**a** to **d**). Source data are provided as a Source Data file.

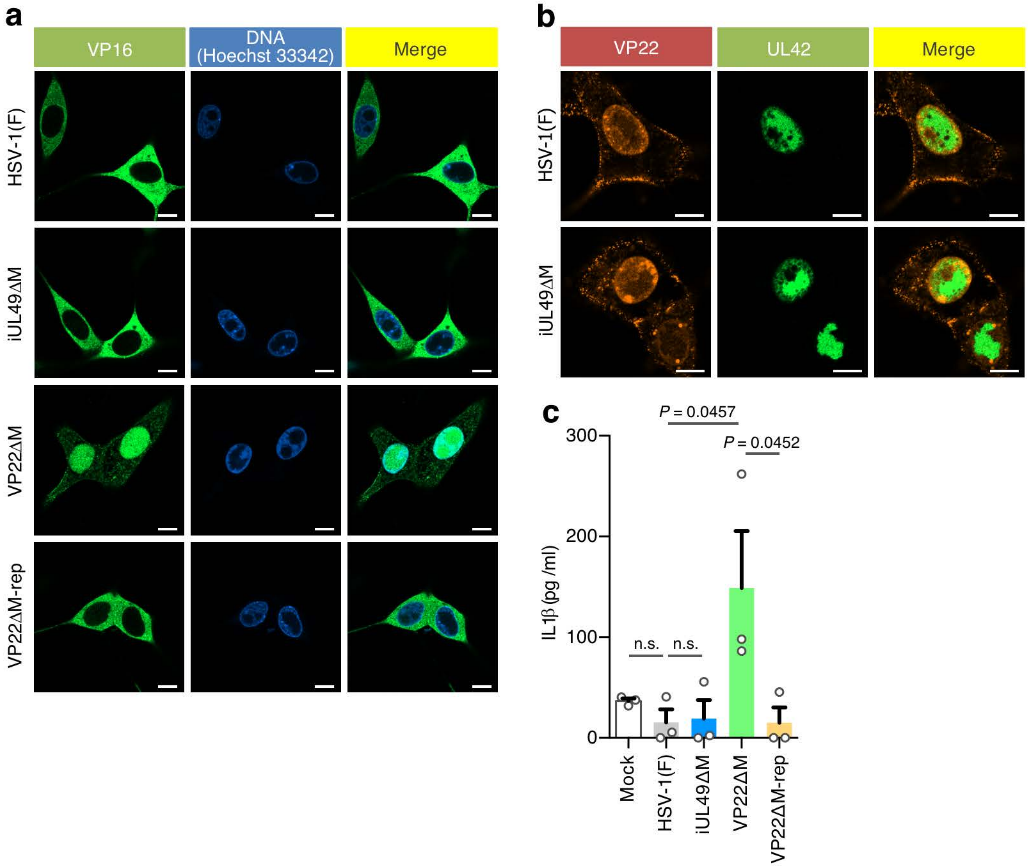


**Supplementary Fig. 10. Effects of piUL49 on VP22-mediated functions. a,** Confocal microscope images of Vero cells infected with wild-type HSV-1(F), iUL49ΔM, VP22ΔM or VP22ΔM-rep for 12 h at an MOI of 10 and stained with Hoechst 33342 or antibody to VP16. Scale bar, 10 µm. **b,** Confocal microscope images of Vero cells infected with wild-type HSV-1(F) or iUL49ΔM for 12 h at an MOI of 10 and stained with antibodies to VP22 and UL42 (Viral DNA polymerase processivity factor). Scale bar, 10 µm. **c,** J774.A1 cells were mock-infected or infected with wild-type HSV-1(F), iUL49ΔM, VP22ΔM or VP22ΔM-rep for 18 h at an MOI of 3. The release of IL-1β into the supernatants was analyzed by ELISA. Each value represents the mean ± SEM of three independent experiments. The indicated *P*-values were obtained using one-way ANOVA followed by Tukey's test. n.s., not significant. Digital images are representative of three independent experiments (**a, b**). Source data are provided as a Source Data file.

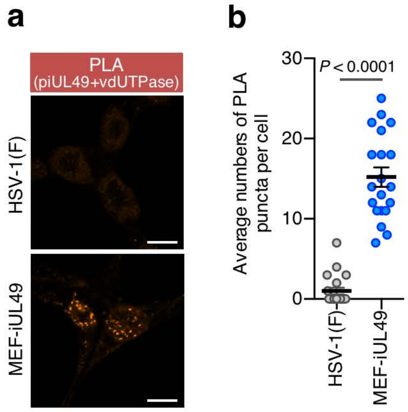


**Supplementary Fig. 11. Physical proximity between MEF-piUL49 and vdUTPase.** Confocal microscope images (**a**) and quantitation (**b**) of the Duolink signaling of vdUTPase and MEF-piUL49 in Vero cells infected with wild-type HSV-1(F) or MEF-iUL49 for 12 h at an MOI of 10. Data are the mean ± SEM (n = 20 for HSV-1-infected and 20 for MEF-iUL49-infected cells representative of three independent experiments). Scale bar, 10 µm. The indicated *P*-values were obtained using an unpaired two-tailed Student's *t*-test. Digital images are representative of three independent experiments. Source data are provided as a Source Data file.

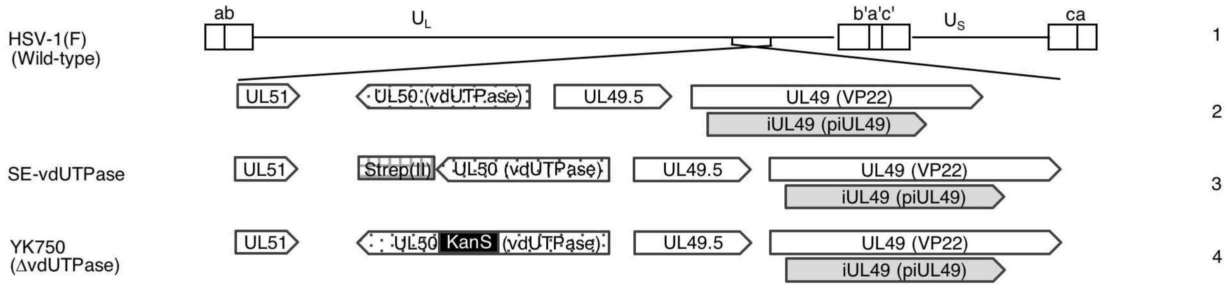


**Supplementary Fig. 12. Genome structure of recombinant viruses-III.** Line 1, the wild-type HSV-1 genome; line 2, the structure of the UL49 (VP22), iUL49 (piUL49), UL49.5, UL50 (vdUTPase) or UL51 CDSs; lines 3, a recombinant virus carrying SE-tagged vdUTPase (SE-vdUTPase); lines 4, a recombinant virus with a mutation in vdUTPase (ΔvdUTPase).

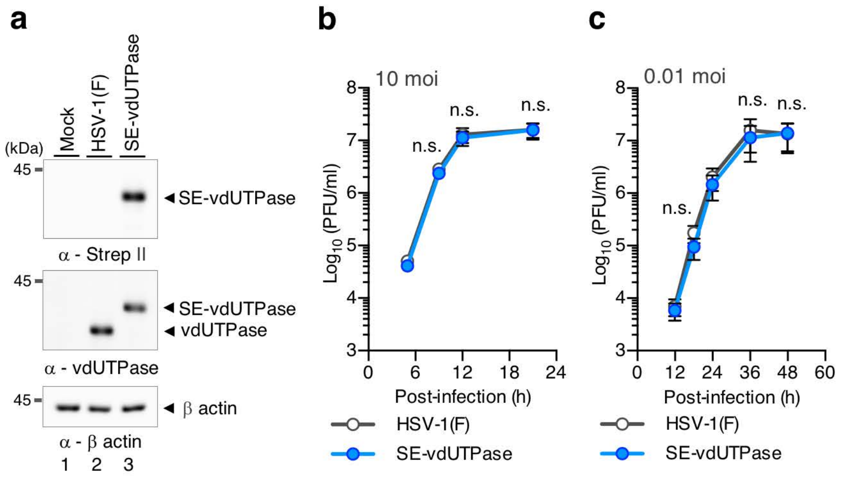


**Supplementary Fig. 13. Tagging of vdUTPase with SE has no effect on HSV-1 replication. a,** Vero cells mock-infected or infected with wild-type HSV-1(F) or SE-vdUTPase for 12 h at an MOI of 10 were lysed and analyzed by immunoblotting with antibodies to Strep-tag II (Strep II), vdUTPase and β actin. **b, c,** Vero cells were infected with HSV-1(F) or SE-vdUTPase at an MOI of 10 (**b**) or 0.01 (**c**). Viral titers in infected cells were assayed as described in **Supplementary Fig. 9**. Each value represents the mean ± SEM of three independent experiments. Each value represents the mean ± SEM of three independent experiments and analyzed by an unpaired two-tailed Student's *t*-test. n.s., not significant. Source data are provided as a Source Data file.

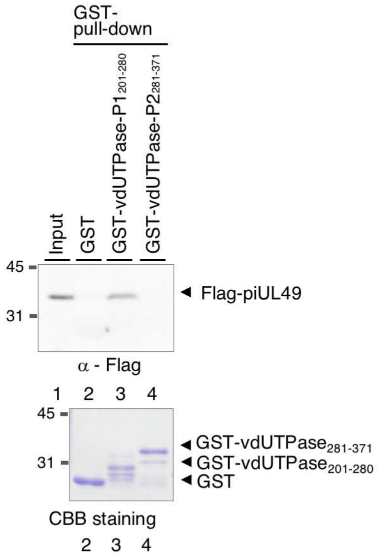


**Supplementary Fig. 14. Binding of vdUTPase to piUL49. a,** GST, GST-vdUTPase$_{201\text{-}280}$ and GST-vdUTPase$_{281\text{-}371}$ immobilized on glutathione-sepharose were reacted with lysates of HEK293FT cells transfected with pFlag-iUL49. The resins were separated on a denaturing gel, transferred onto a nitrocellulose membrane and reacted with antibody to Flag (top) or subjected to CBB staining (bottom). Digital images are representative of three independent experiments.

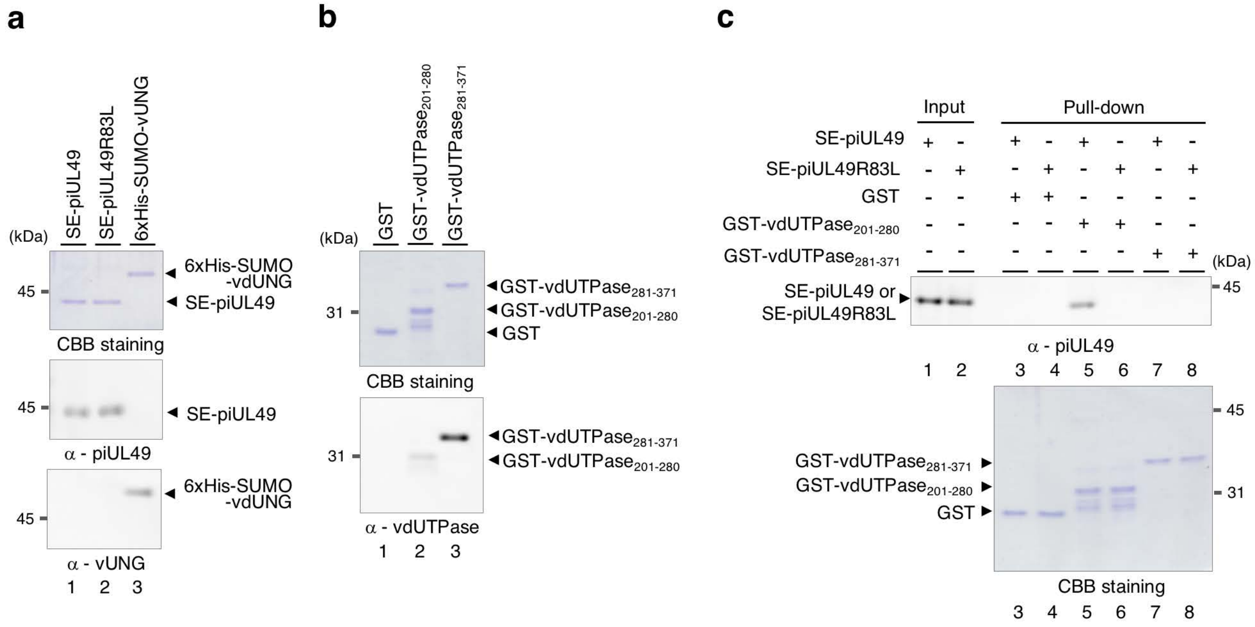


**Supplementary Fig. 15. Direct binding of vdUTPase to piUL49-I. a, b,** Purified SE-piUL49 (**a**), SE-piUL49R83L (**a**), 6xHis-SUMO-vUNG (**a**), GST (**b**), GST-vdUTPase$_{201\text{-}280}$ (**b**) and GST-vdUTPase$_{281\text{-}371}$ (**b**) were separated on a denaturing gels and subjected to CBB staining (top) or transferred onto a nitrocellulose membrane and reacted with antibodies to piUL49 (**a**), vUNG (**a**) or vdUTPase **(b)** (bottom). **c**, GST, GST-vdUTPase$_{201\text{-}280}$ and GST-vdUTPase$_{281\text{-}371}$ immobilized on glutathione-sepharose were reacted with purified SE-piUL49 or SE-piUL49R83L. The resins were separated on a denaturing gel, transferred onto a nitrocellulose membrane, and reacted with antibody to piUL49 (top) or subjected to CBB staining (bottom). Digital images are representative of three independent experiments.

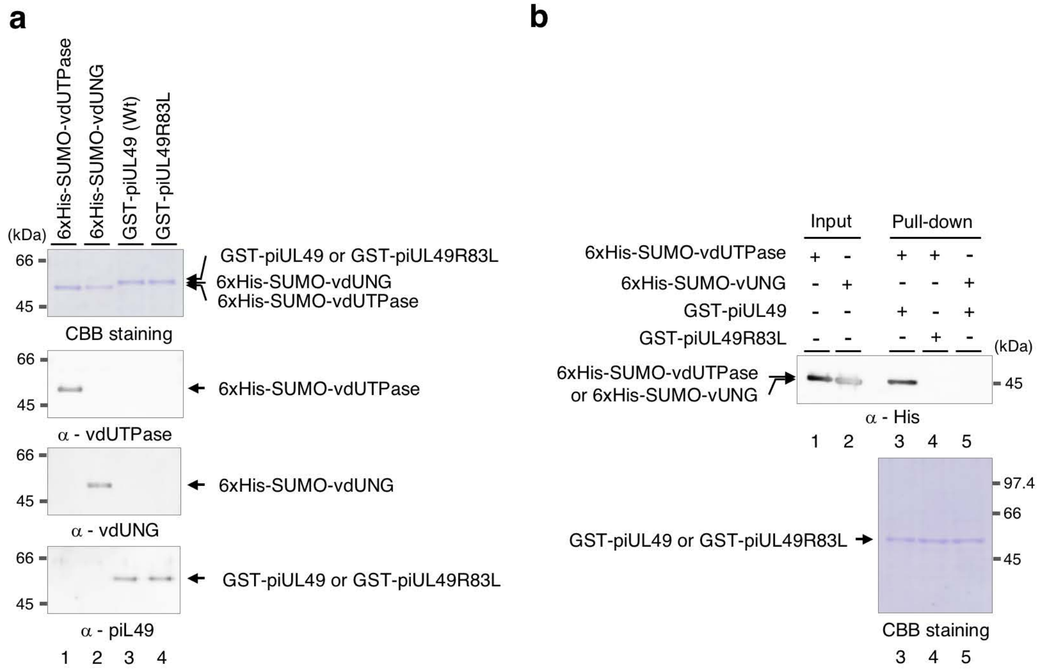


**Supplementary Fig. 16. Direct binding of piUL49 to vdUTPase-II. a,** Purified 6xHis-SUMO-vdUTPase, 6xHis-SUMO-vUNG, GST-piUL49 and GST-piUL49R83L were separated on a denaturing gel and subjected to CBB staining (top column) or transferred onto a nitrocellulose membrane and reacted with antibodies to vdUTPase (second column), vUNG (third column) and piUL49 (bottom column). **b,** GST-piUL49 and GST-piUL49R83L immobilized on glutathione-sepharose were reacted with purified 6xHis-SUMO-vdUTPase, and 6xHis-SUMO-vUNG, respectively. The resins were separated on a denaturing gel, transferred onto a nitrocellulose membrane, and reacted with antibody to His-tag (top) or subjected to CBB staining (bottom). Digital images are representative of three independent experiments.

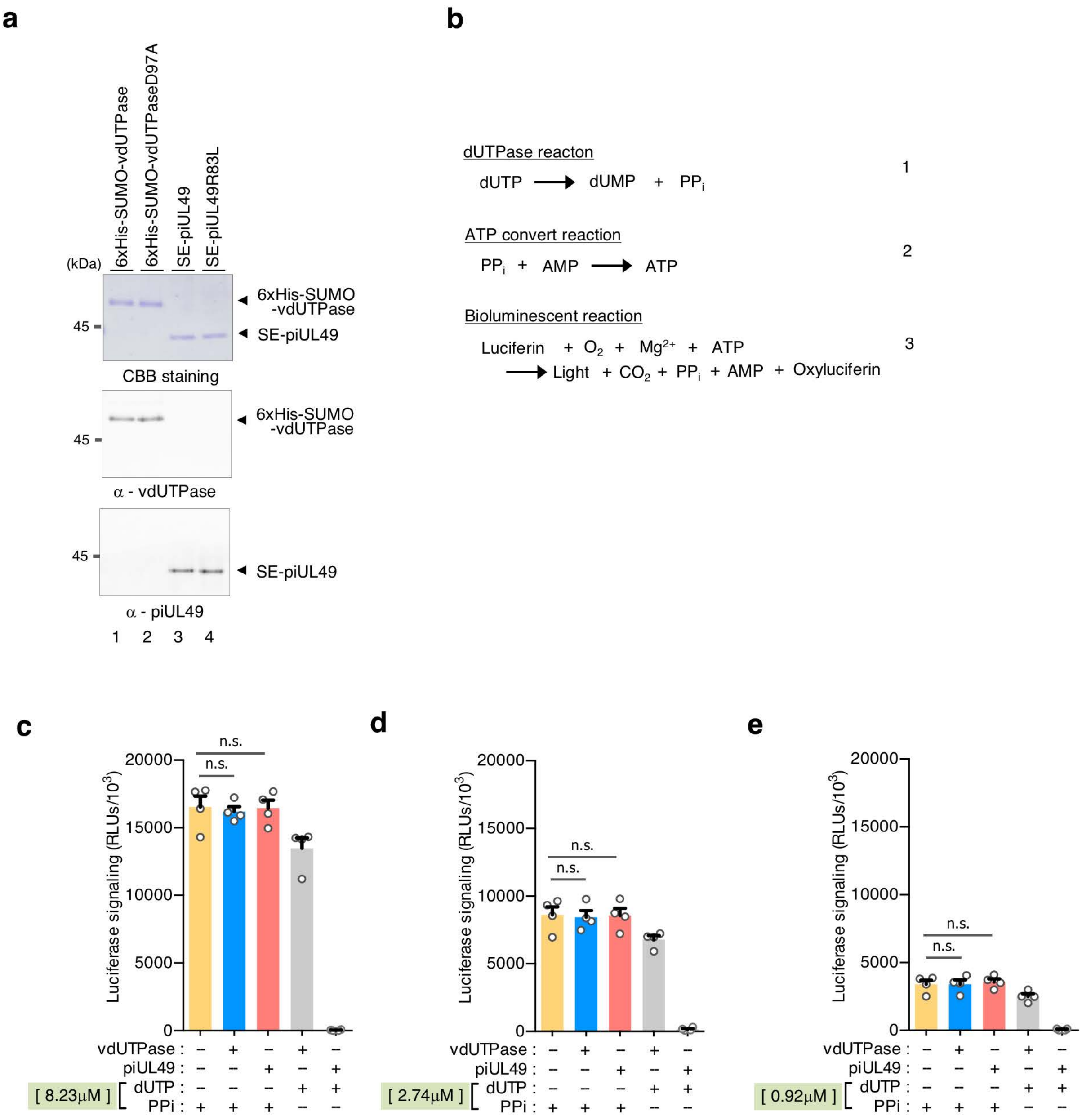
a
6xHis-SUMO-vdUTPase
6xHis-SUMO-vdUTPaseD97A
SE-piUL49
SE-piUL49R83L
(kDa)
45
6xHis-SUMO -vdUTPase
SE-piUL49
CBB staining
45
6xHis-SUMO -vdUTPase
α - vdUTPase
45
SE-piUL49
α - piUL49
1 2 3 4
b
dUTPase reacton
dUTP → dUMP + PPi 1
ATP convert reaction
PPi + AMP → ATP 2
Bioluminescent reaction
Luciferin + O2 + Mg2+ + ATP 3
→ Light + CO2 + PPi + AMP + Oxyluciferin
c
d
e
Luciferase signaling (RLUs/10^3)
20000
15000
10000
5000
0
n.s.
n.s.
vdUTPase : − + − + −
piUL49 : − − + − +
dUTP : − − − + +
PPi : + + + − −
[ 8.23μM ]
[ 2.74μM ]
[ 0.92μM ]

**Supplementary Fig. 17. Effect of vdUTPase or piUL49 on ATP conversion and bioluminescent reactions in a dUTPase assay. a,** Purified 6xHis-SUMO-vdUTPase, 6xHis-SUMO-vdUTPaseD97A, SE-piUL49 and SE-piUL49R83L were separated on a denaturing gel and subjected to CBB staining (top) or transferred onto a nitrocellulose membrane and reacted with antibodies to vdUTPase (middle) or piUL49 (bottom). **b,** Scheme of vdUTPase activity detection. Line 1, viral dUTPase hydrolyzes dUTP to dUMP and PPi; line 2, the converting reagent catalyzes the conversion of AMP and the enzymatically-produced PPi to ATP; line 3, luciferase of detection reagent produces light from the newly formed ATP and luciferin. **c to e,** Efficiency of ATP conversion (**b**, line 2) and bioluminescent (**b**, line 3) reactions in the absence or presence of purified 0.3 μM 6xHis-SUMO-vdUTPase or SE-piUL49, measured by luciferase signaling. Relative luminescence units (RLUs) were normalized by subtracting the value of each reaction from that of vdUTPaseD97A. The purified proteins had no effect on the efficiency of ATP conversion and bioluminescent reactions without dUTP (orange, blue and red bars). Bioluminescence signals for 6 h reactions in the presence of 0.3 μM 6xHis-SUMO-vdUTPase and 0.92 (**c**), 2.74 (**d**) or 8.23 (**e**) μM dUTP (gray bars) were comparable to those for 6 h mock-reactions followed by 0.92 (**c**), 2.74 (**d**) or 8.23 (**e**) μM PPi addition in the absence of dUTP (orange bars), respectively. Each value represents the mean ± SEM of four independent experiments and analyzed by one-way ANOVA followed by Tukey's test. n.s., not significant. Source data are provided as a Source Data file.

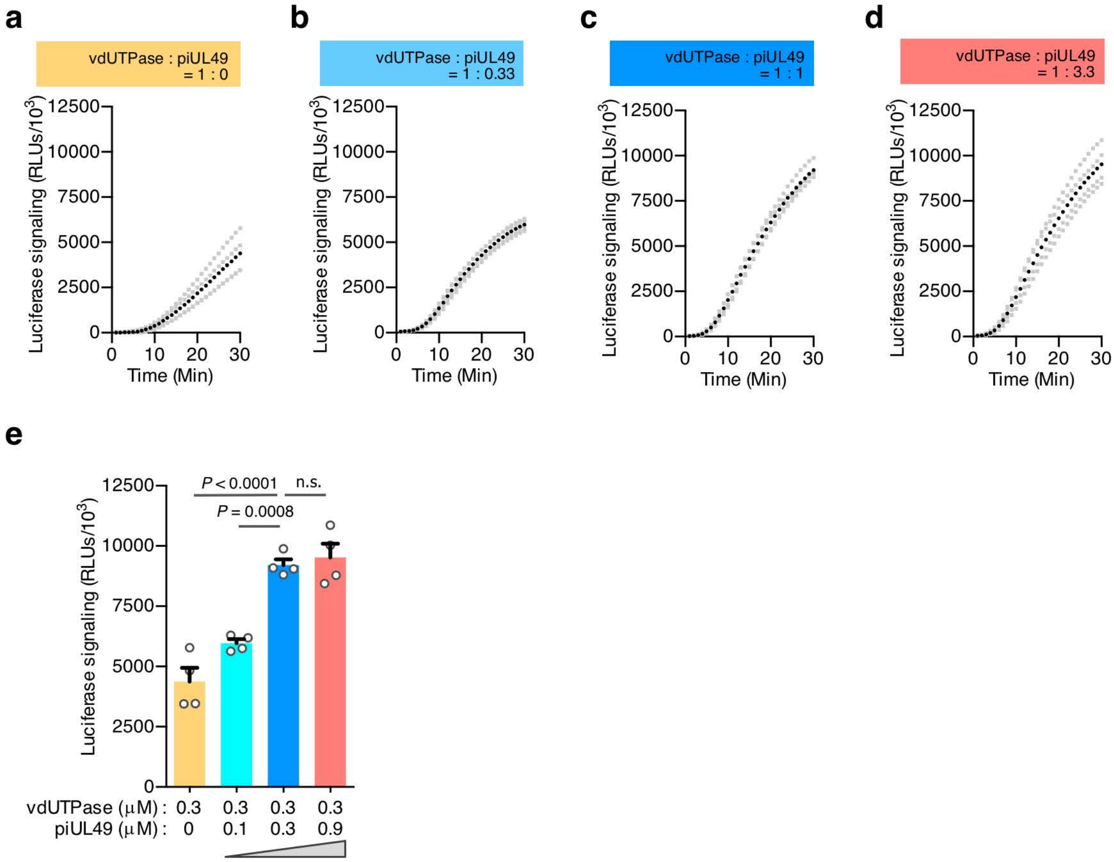


**Supplementary Fig. 18. Effect of piUL49 concentration on vdUTPase activity. a to d,** In the absence or presence of purified 0.1, 0.3 or 0.9 μM SE-piUL49, 0.3 μM 6xHis-SUMO-vdUTPase was mixed with 74.07 μM dUTP. The release of PPi upon dUTP hydrolysis by 6xHis-SUMO-vdUTPase was measured as luciferase signaling and RLUs were normalized by subtracting the value of vdUTPase from that of vdUTPaseD97A. A black spot indicates the mean ± SEM of four independent experiments and a gray spot indicates the results of individual experiments. **e.** vdUTPase activity based on (**a** to **d**). Each value represents the mean ± SEM of four independent experiments. The indicated *P*-values were obtained using one-way ANOVA followed by Tukey's test. n.s., not significant. Source data are provided as a Source Data file.

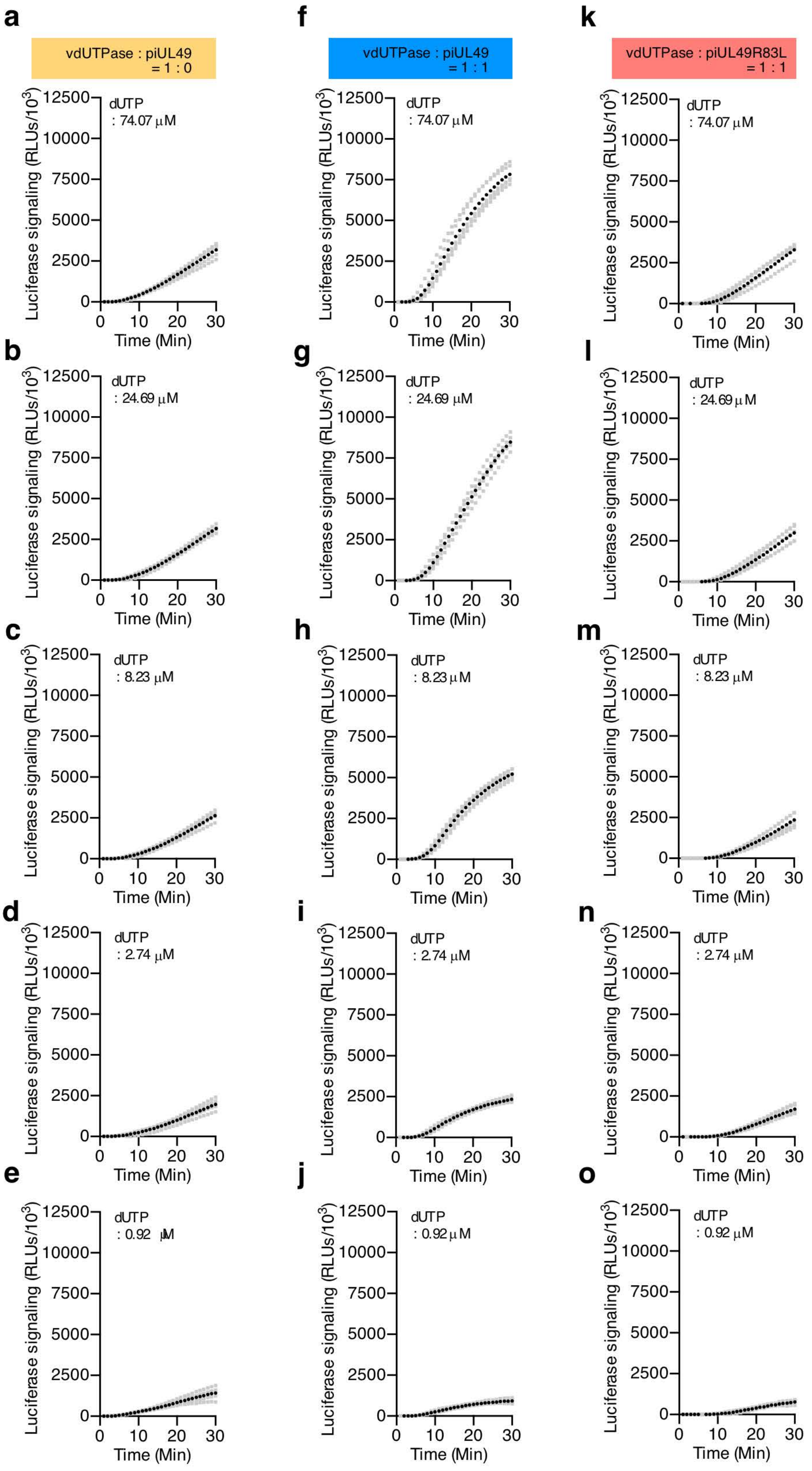
a
vdUTPase : piUL49 = 1 : 0
f
vdUTPase : piUL49 = 1 : 1
k
vdUTPase : piUL49R83L = 1 : 1
b
g
l
c
h
m
d
i
n
e
j
o
dUTP : 74.07 µM
dUTP : 24.69 µM
dUTP : 8.23 µM
dUTP : 2.74 µM
dUTP : 0.92 µM
Luciferase signaling (RLUs/10^3)
Time (Min)

**Supplementary Fig. 19. Kinetic analyses of vdUTPase activities. a to o,** In the absence or presence of purified 0.3 μM SE-piUL49 or SE-piUL49R83L, 0.3 μM 6xHis-SUMO-vdUTPase was mixed with 0.92, 2.74, 8.23, 24.69 or 74.07 μM dUTP. The release of PPi upon dUTP hydrolysis by 6xHis-SUMO-vdUTPase was measured as luciferase signaling and RLUs were normalized by subtracting the value of vdUTPase from that of vdUTPaseD97A. A black spot indicates the mean ± SEM of five independent experiments and a gray spot indicates the results of individual experiments. Source data are provided as a Source Data file.

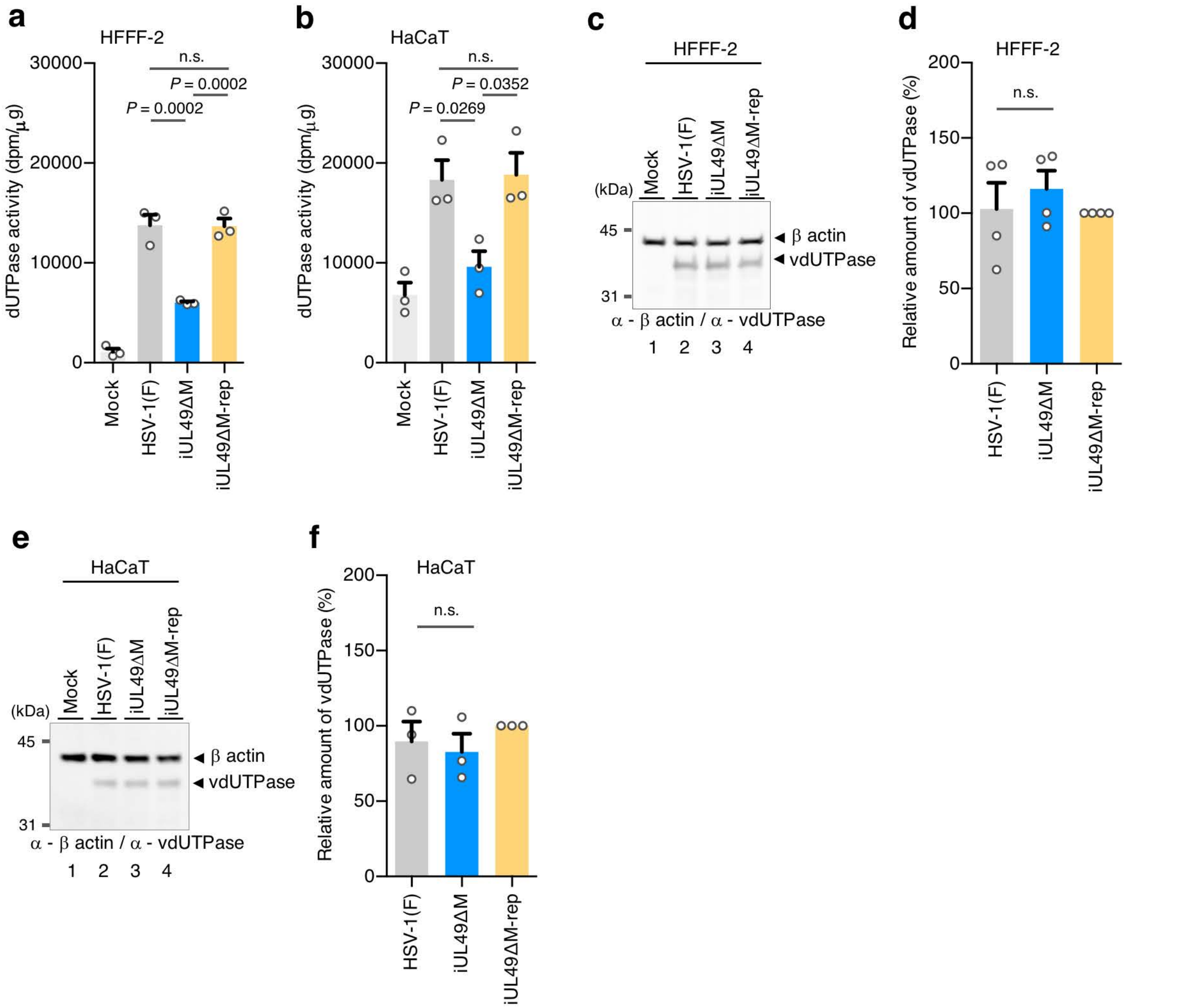


**Supplementary Fig. 20. Effect of piUL49 on dUTPase activity in infected human cells. a, b,** dUTPase activities (dpm/μg of total protein) of HFFF-2 (**a**) or HaCaT (**b**) cells infected with HSV-1(F), iUL49ΔM or iUL49ΔM-rep for 12 h at an MOI of 10. Each value is the mean ± SEM of thee independent experiments. The indicated *P*-values were obtained using one-way ANOVA followed by Tukey's test. n.s., not significant. **c, e,** HFFF-2 (**c**) or HaCaT (**e**) cells mock-infected or infected with wild-type HSV-1(F), iUL49ΔM or iUL49ΔM-rep for 12 h at an MOI of 10 were lysed and analyzed by immunoblotting with antibodies to vdUTPase and β actin. Digital images are representative of four (**c**) or three (**e**) independent experiments. **d, f,** The amounts of vdUTPase proteins were quantitated and normalized to those of β actin proteins. Each value is the mean ± SEM of four (**d**) or three (**f**) independent experiments and is expressed relative to that in cells infected with iUL49ΔM-rep, which was normalized to 100%. Each values were analyzed by an unpaired two-tailed Student's *t*-test. n.s., not significant. Source data are provided as a Source Data file.

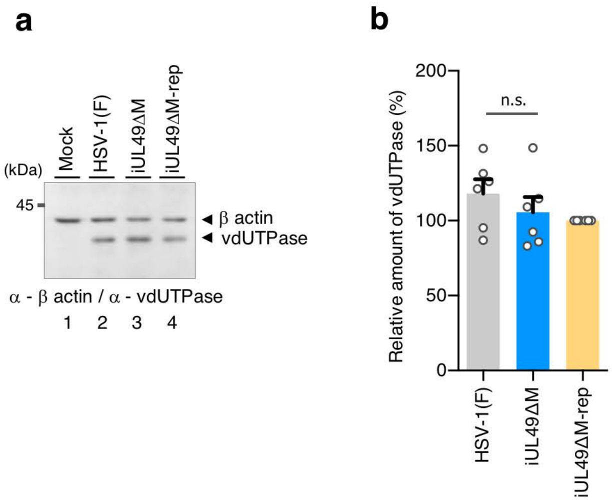


**Supplementary Fig. 21. piUL49 has no effect on accumulation of vdUTPase. a,** Vero cells mock-infected or infected with wild-type HSV-1(F), iUL49ΔM or iUL49ΔM-rep for 12 h at an MOI of 10 were lysed and analyzed by immunoblotting with antibodies to vdUTPase and β actin. **b,** The amounts of vdUTPase proteins in the experiment in (**a**) were quantitated and normalized to those of β actin proteins. Each value is the mean ± SEM of six independent experiments and is expressed relative to that in cells infected with iUL49ΔM-rep, which was normalized to 100%. Each values were analyzed by an unpaired two-tailed Student's *t*-test. n.s., not significant. Source data are provided as a Source Data file.

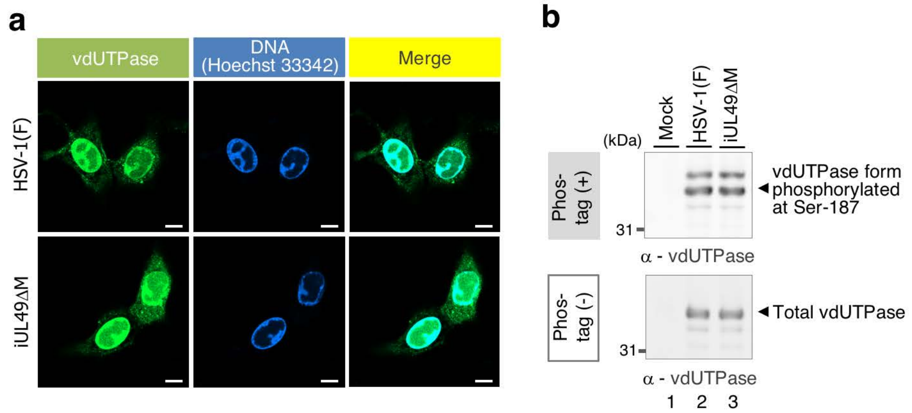


**Supplementary Fig. 22. Effects of piUL49 on vdUTPase localization and phosphorylation. a**, Confocal microscope images of Vero cells mock-infected or infected with wild-type HSV-1(F) or iUL49ΔM for 12 h at an MOI of 10 and stained with Hoechst 33342 or antibodies to vdUTPase. Scale bar, 10 μm. **b,** Vero cells mock-infected or infected with wild-type HSV-1(F) or iUL49ΔM for 12 h at an MOI of 10 were lysed and analyzed on a Phos-tag(+) SDS-PAGE gel (top) or Phos-tag(−) SDS-PAGE gel (bottom). The gels were immunoblotted with antibodies to vdUTPase. Digital images are representative of three independent experiments.

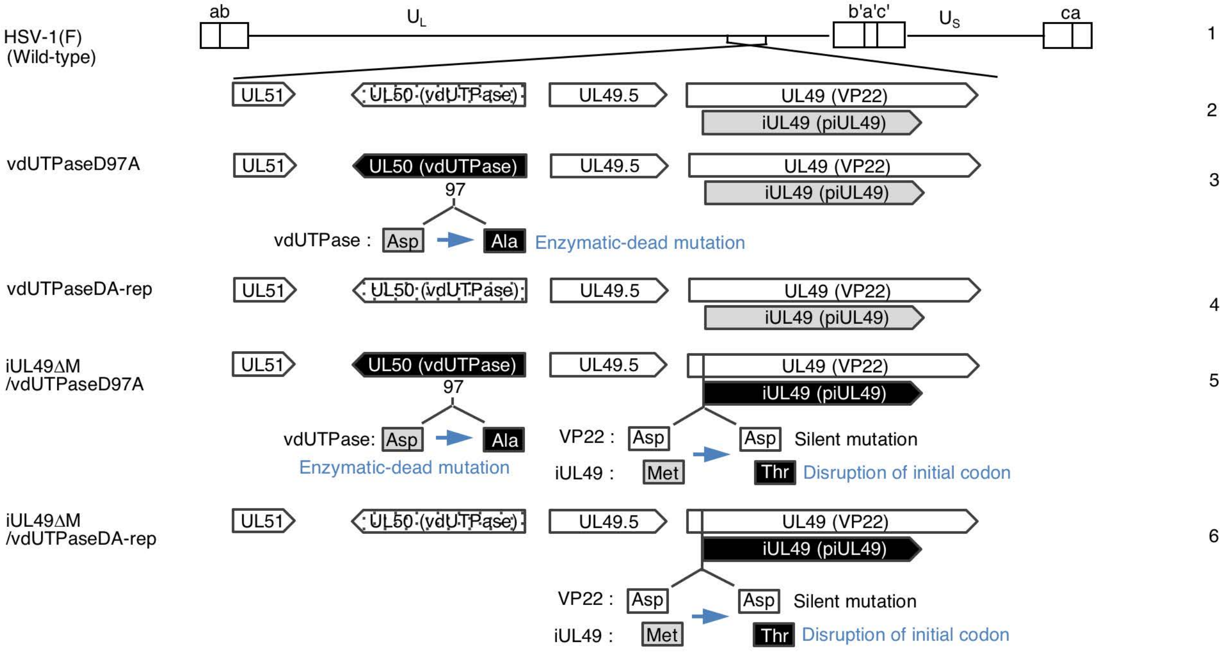


**Supplementary Fig. 23. Genome structure of recombinant viruses-IV.** Line 1, the wild-type HSV-1 genome; line 2, the structure of the UL49 (VP22), iUL49 (piUL49), UL49.5, UL50 (vdUTPase) or UL51 CDSs; lines 3 to 6, recombinant viruses with a mutation(s) in iUL49 and/or UL50 (vdUTPase). All substitutions in the iUL49 gene shown in lines 3 and 5 were designed to have no effect on the amino acid sequence of VP22.

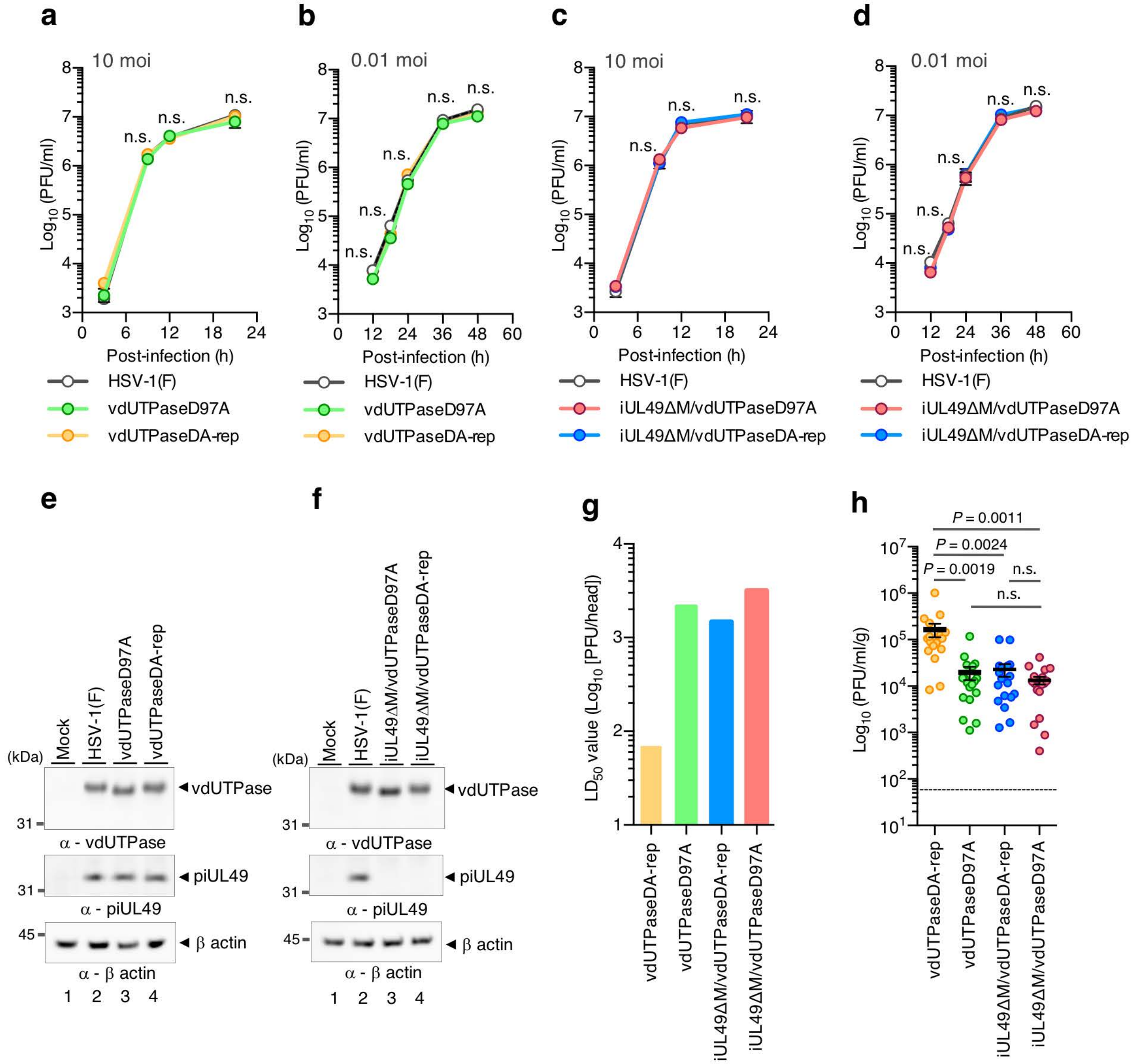


**Supplementary Fig. 24. Effects of D97A mutation in vdUTPase on the iUL49-null mutant. a to d,** Vero cells were infected with wild-type HSV-1(F) (**a** to **d**), vdUTPaseD97A (**a** and **b**), vdUTPaseDA-rep (**a** and **b**), iUL49ΔM/vdUTPaseD97A (**c** and **d**), or iUL49ΔM/vdUTPaseDA-rep (**c** and **d**) at an MOI of 10 (**a** and **c**) or 0.01 (**b** and **d**). Viral titers in infected cells were assayed as described in **Supplementary Fig. 9**. Each value represents the mean ± SEM of three independent experiments and analyzed by one-way ANOVA followed by Tukey's test. n.s., not significant. **e, f,** Vero cells mock-infected or infected with HSV-1(F) vdUTPaseD97A (**e**), vdUTPaseDA-rep (**e**), iUL49ΔM/vdUTPaseD97A (**f**) or iUL49ΔM/vdUTPaseDA-rep (**f**) for 12 h at an MOI of 10 were lysed and analyzed by immunoblotting with antibodies to vdUTPase, piUL49 and β actin. Digital images are representative of three independent experiments (**e**, **f**). **g,** $LD_{50}$ values of mice intracranially infected with vdUTPaseDA-rep, vdUTPaseD97A, iUL49ΔM/vdUTPaseDA-rep or iUL49ΔM/vdUTPaseD97A were determined as described in **Fig. 3i to k.** **h**, Eighteen 3-week-old female ICR mice were infected intracranially with 100 PFU/head of vdUTPaseDA-rep, vdUTPaseD97A, iUL49ΔM/vdUTPaseDA-rep or iUL49ΔM/vdUTPaseD97A. Viral titers in mouse brains at 3 d post-infection were assayed. Dashed line indicates the limit of detection. Each value is the mean ± SEM for each group. The indicated *P*-values were obtained using one-way ANOVA followed by Tukey's test. n.s., not significant. Source data are provided as a Source Data file.

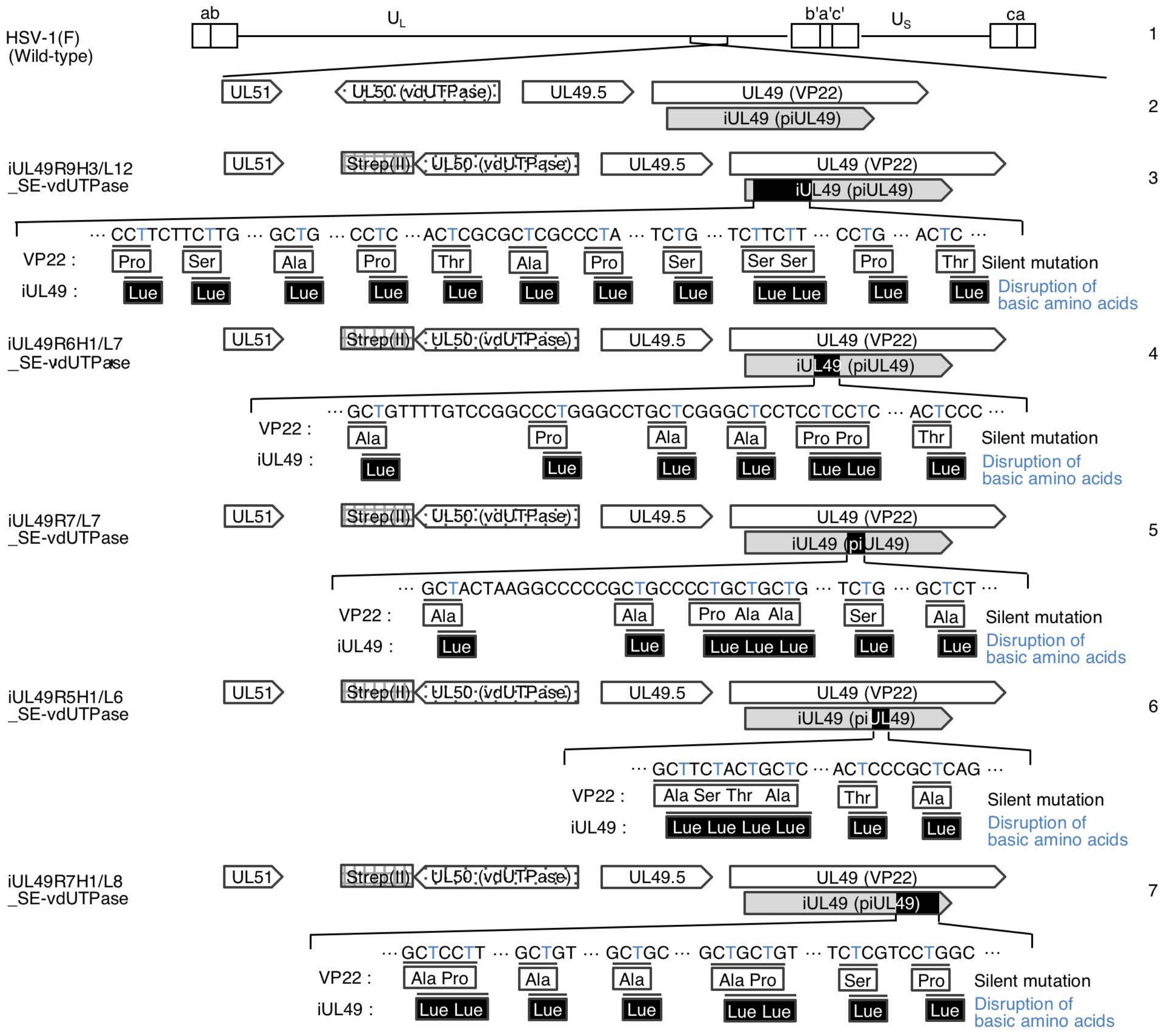


**Supplementary Fig. 25. Genome structure of recombinant viruses-V.** Line 1, the wild-type HSV-1 genome; line 2, the structure of the UL49 (VP22), iUL49 (piUL49), UL49.5, UL50 (vdUTPase) or UL51 CDSs; lines 3 to 7, recombinant viruses with mutations in iUL49 and carrying SE-vdUTPase. All substitutions in the iUL49 gene shown in lines 3 to 7 were designed to have no effect on the amino acid sequence of VP22.

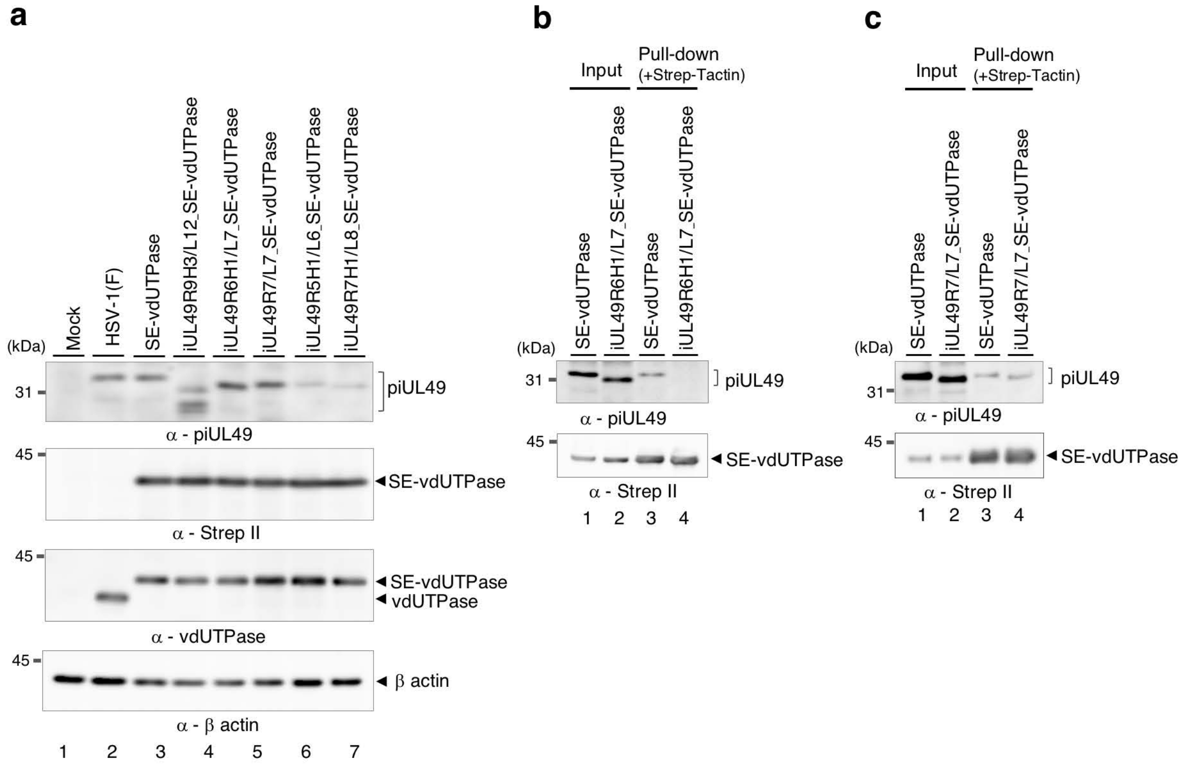


**Supplementary Fig. 26. Effects of mutations in piUL49 on its binding to vdUTPase. a,** Vero cells mock-infected or infected with wild-type HSV-1(F), SE-vdUTPase, iUL49R9H3/L12_SE-vdUTPase, iUL49R6H1/L7_SE-vdUTPase, iUL49R7/L7_SE-vdUTPase, iUL49R5H1/L6_SE-vdUTPase or iUL49R7H1/L8_SE-vdUTPase for 12 h at an MOI of 10 were lysed and analyzed by immunoblotting with antibodies to piUL49, Strep II, vdUTPase and β actin. **b, c,** Vero cells were infected with SE-vdUTPase (**b, c**), iUL49R6H1/L7_SE-vdUTPase (**b**) or iUL49R7/L7_SE-vdUTPase (**c**) for 12 h at an MOI of 10, lysed, precipitated with StrepTactin-sepharose and analyzed by immunoblotting with antibodies to iUL49 and Strep II. Digital images are representative of three independent experiments.

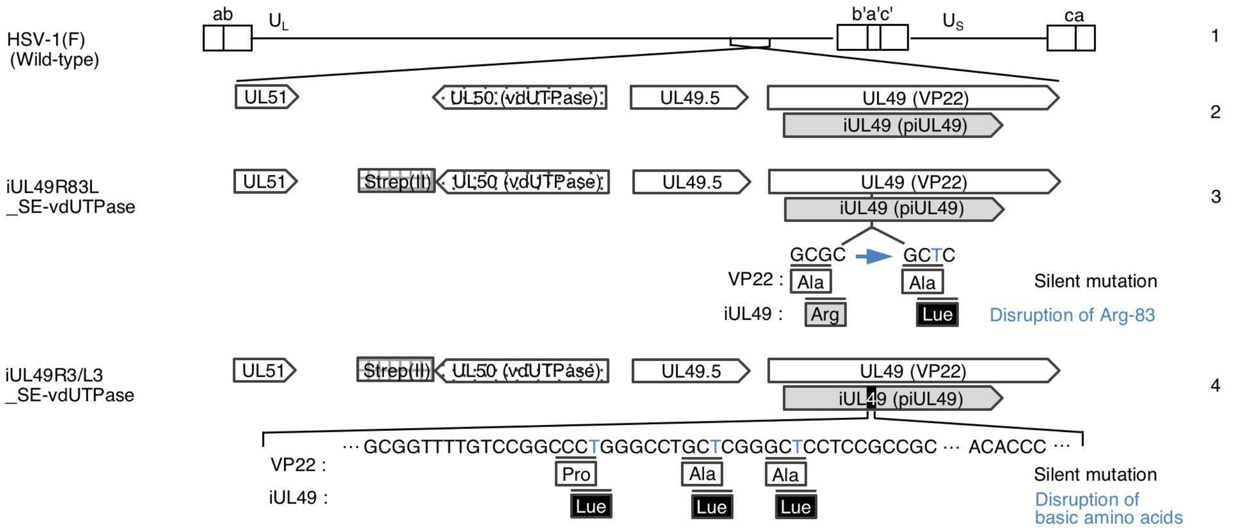


**Supplementary Fig. 27. Genome structure of recombinant viruses-VI.** Line 1, the wild-type HSV-1 genome; line 2, the structure of the UL49 (VP22), iUL49 (piUL49), UL49.5, UL50 (vdUTPase) or UL51 CDSs; lines 3 and 4, recombinant viruses with a mutation(s) in iUL49 and carrying SE-vdUTPase. All substitutions in the iUL49 gene shown in lines 3 and 4 were designed to have no effect on the amino acid sequence of VP22.

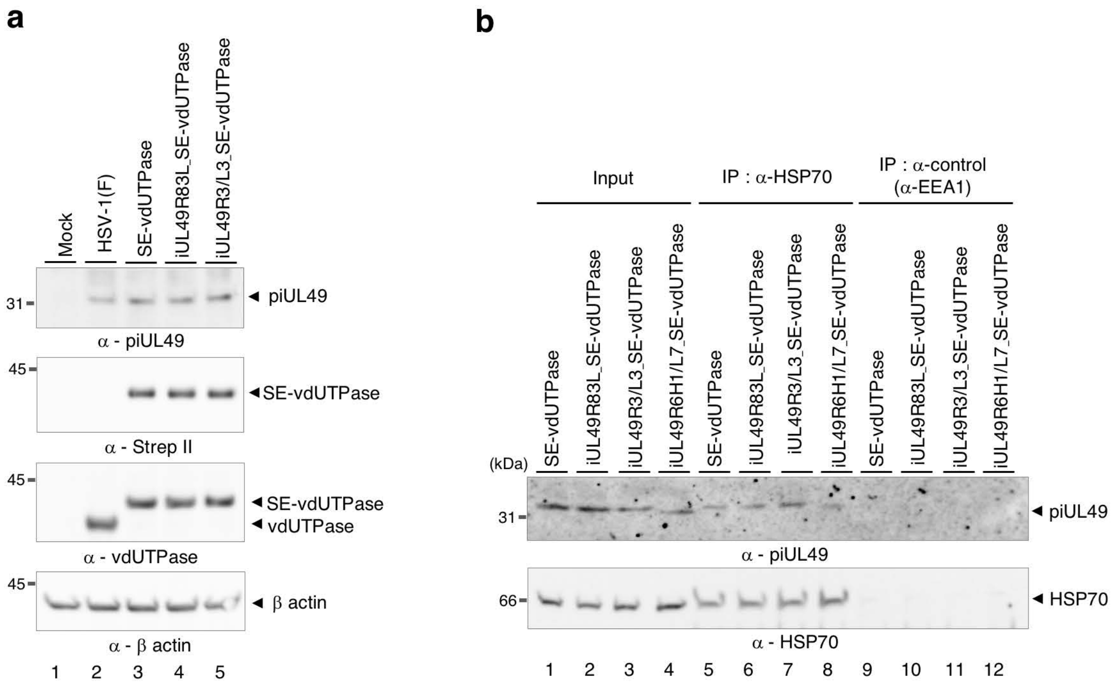


**Supplementary Fig. 28. Effects of mutations in piUL49 on its binding to HSP70. a,** Vero cells mock-infected or infected with wild-type HSV-1(F), SE-vdUTPase, iUL49R83L_SE-vdUTPase or iUL49R3/L3_SE-vdUTPase for 12 h at an MOI of 10 were lysed and analyzed by immunoblotting with antibodies to piUL49, Strep II, vdUTPase and β actin. **b**, Vero cells infected with SE-vdUTPase, iUL49R83L_SE-vdUTPase, iUL49R3/L3_SE-vdUTPase or iUL49R6H1/L7_SE-vdUTPase for 12 h at an MOI of 10, were lysed, immunoprecipitated with anti-HSP70 antibody or unrelated control antibody (anti-EEA1 antibody) and analyzed by immunoblotting with antibodies to iUL49 and HSP70. Digital images are representative of three independent experiments.

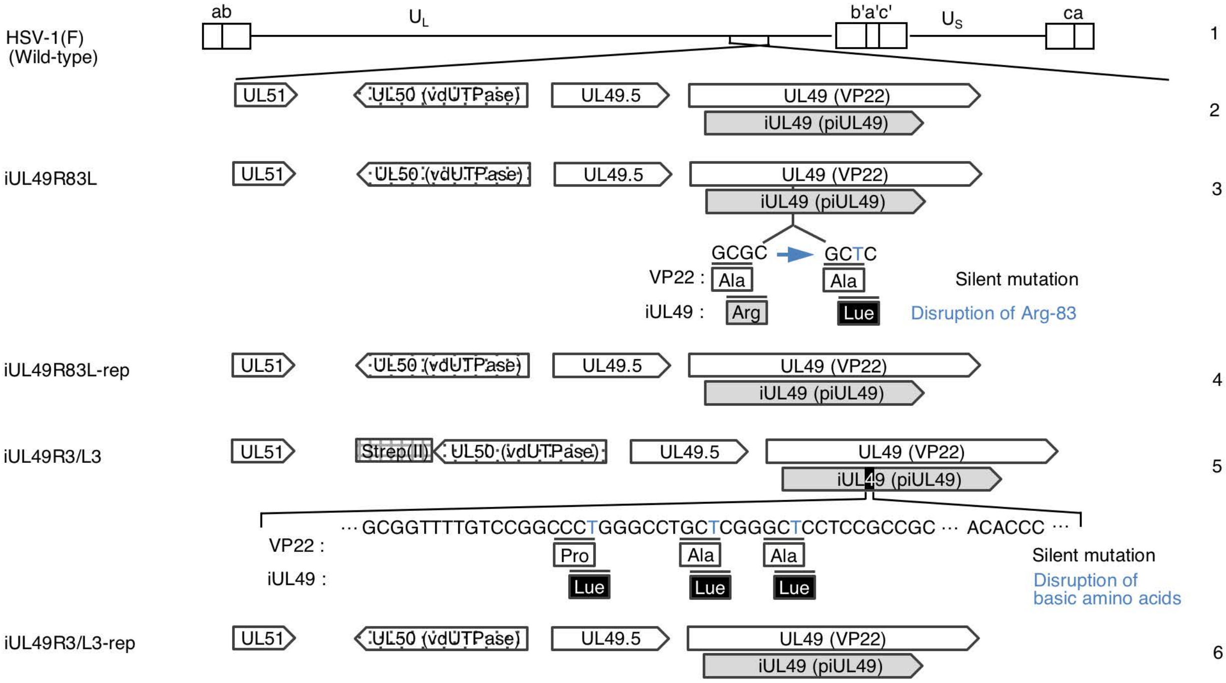


**Supplementary Fig. 29. Genome structure of recombinant viruses-VII.** Line 1, the wild-type HSV-1 genome; line 2, the structure of the UL49 (VP22), iUL49 (piUL49), UL49.5, UL50 (vdUTPase) or UL51 CDSs; lines 3 to 6, recombinant viruses with a mutation(s) in iUL49. All substitutions in the iUL49 gene shown in lines 3 and 5 were designed to have no effect on the amino acid sequence of VP22.

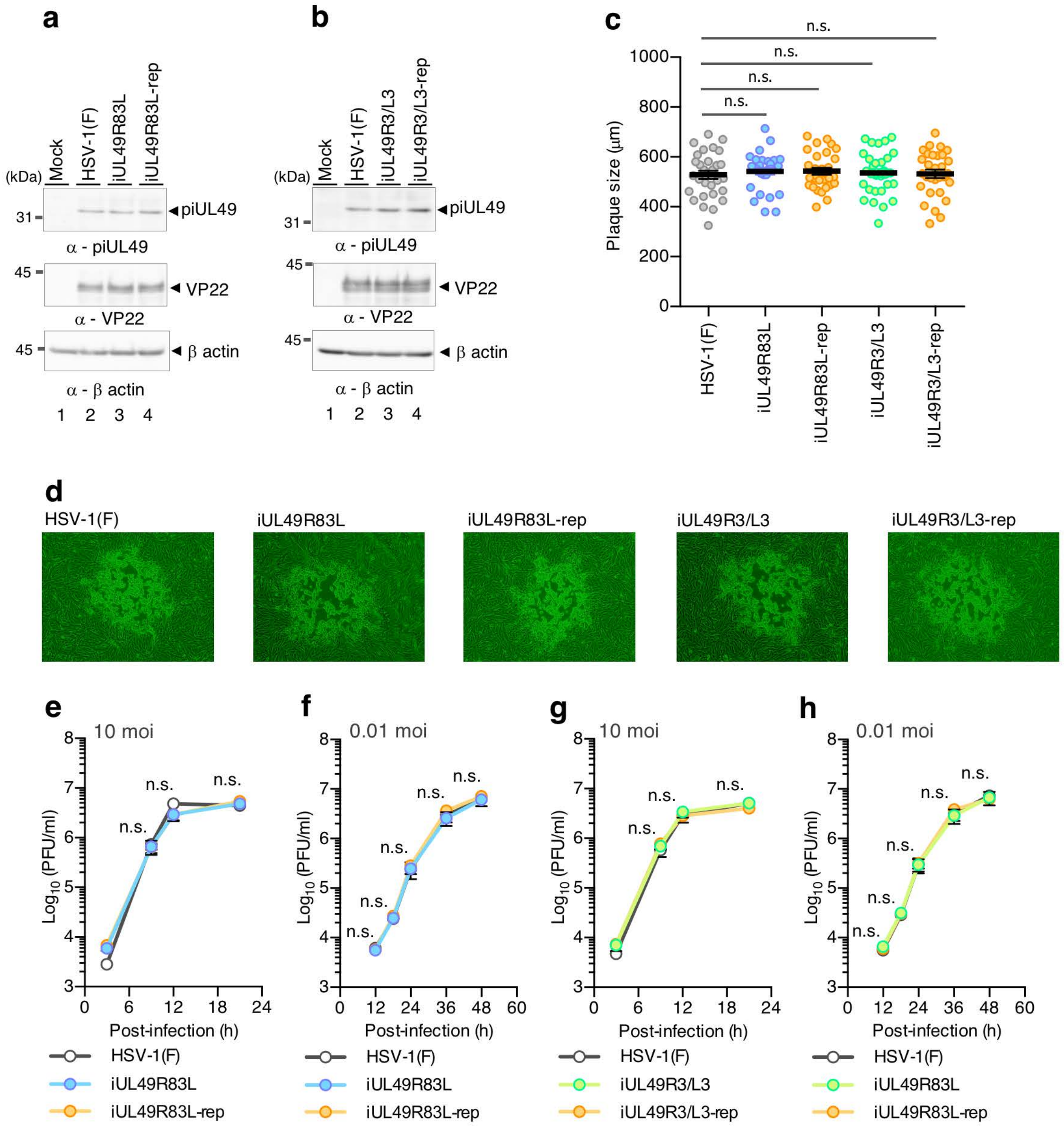


**Supplementary Fig. 30. iUL49R83L mutation has no effect on HSV-1 replication. a, b,** Vero cells mock-infected (**a, b**) or infected with wild-type HSV-1(F) (**a, b**), iUL49R83L (**a**), iUL49R83L-rep (**a**), iUL49R3/L3 (**b**) or iUL49R3/L3-rep (**b**) for 12 h at a MOI of 10 were lysed and analyzed by immunoblotting with antibodies to piUL49, VP22 and β actin. Digital images are representative of three (**a, b**) independent experiments. **c, d,** Thirty plaque diameters (**c**) and representative images (**d**) of Vero cells infected with HSV-1(F), iUL49R83L, iUL49R83L-rep, iUL49R3/L3 or iUL49R3/L3-rep were measured at 48 h post-infection. Scale bar, 200 μm. Each value is the mean ± SEM for each group and analyzed by one-way ANOVA followed by Tukey's test. n.s., not significant. **e to h,** Vero cells were infected with HSV-1(F) (**e to h**), iUL49R83L (**e, f**), iUL49R83L-rep (**e, f**), iUL49R3/L3 (**g, h**) or iUL49R3/L3-rep (**g, h**) at an MOI of 10 (**e, g**) or 0.01 (**f, h**). Viral titers in infected cells were assayed as described in **Supplementary Fig. 9**. Each value represents the mean ± SEM of three (**e to h**) independent experiments and analyzed by one-way ANOVA followed by Tukey's test. n.s., not significant. Source data are provided as a Source Data file.

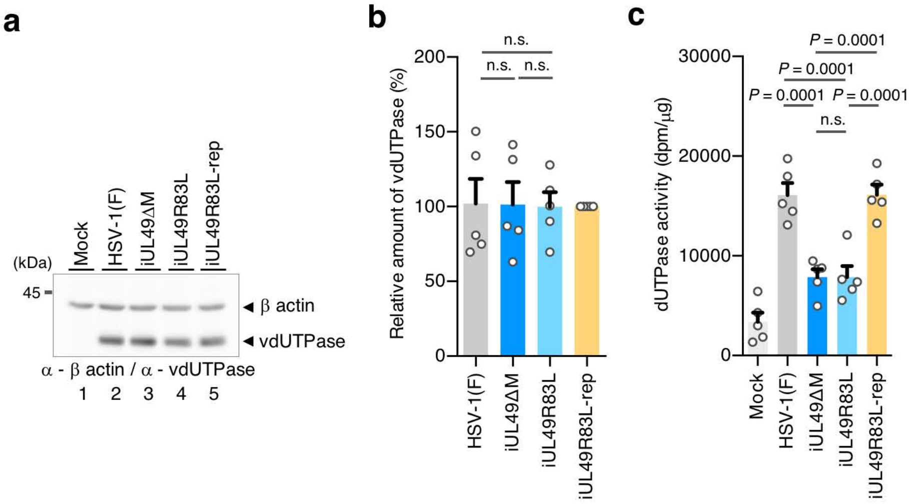


**Supplementary Fig. 31. iUL49R83L mutation reduces dUTPase activity. a,** Vero cells mock-infected or infected with wild-type HSV-1(F), iUL49ΔM, iUL49R83L or iUL49R83L-rep for 12 h at an MOI of 10 were lysed and analyzed by immunoblotting with antibodies to vdUTPase and β actin. Digital images are representative of five (**a**) independent experiments. **b,** The amounts of vdUTPase proteins were quantitated and normalized to those of β actin proteins. Each value is the mean ± SEM of five independent experiments and is expressed relative to that in cells infected with iUL49 R83L-rep, which was normalized to 100%. Each values were analyzed by one-way ANOVA followed by Tukey's test. n.s., not significant. **c,** dUTPase activities (dpm/μg of total protein) of Vero cells infected with HSV-1(F), iUL49ΔM, iUL49R83L or iUL49R83L-rep for 12 h at an MOI of 10. Each value is the mean ± SEM of five independent experiments. The indicated *P*-values were obtained using one-way ANOVA followed by Tukey's test. n.s., not significant. Source data are provided as a Source Data file.

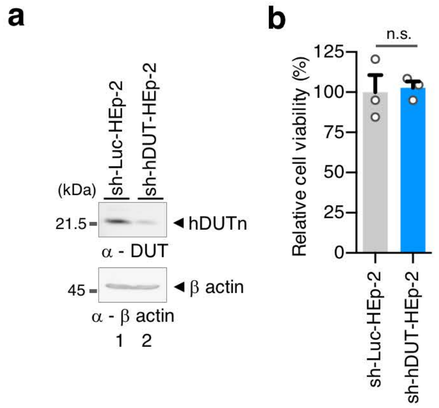


**Supplementary Fig. 32. Characterization of sh-hDUT-HEp-2 cells. a,** sh-Luc-HEp-2 or sh-hDUT-HEp-2 were lysed and analyzed by immunoblotting with antibodies to human dUTPase (hDUT) or β actin. Digital images are representative results of three independent experiments. **b,** Cell viability of sh-Luc-HEp-2 and sh-Luc-hDUT-HEp-2 cells. Each value is the mean ± SEM of the results of three independent experiments and is expressed relative to the mean for sh-Luc-HEp-2 cells, which was normalized to 100%. Each values were analyzed by an unpaired two-tailed Student's *t*-test. n.s., not significant. Source data are provided as a Source Data file.

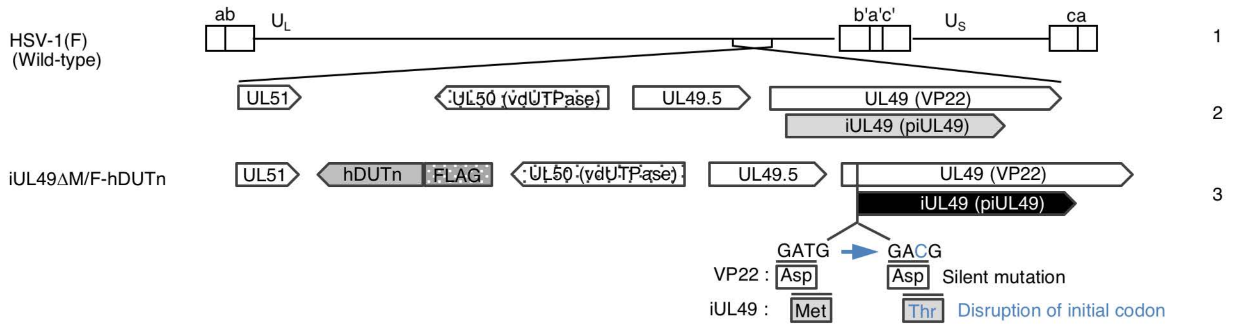


**Supplementary Fig. 33. Genome structure of recombinant viruses-VIII.** Line 1, the wild-type HSV-1 genome; line 2, the structure of the UL49 (VP22), iUL49 (piUL49), UL49.5, UL50 (vdUTPase) or UL51 CDSs; line 3, a recombinant virus with a mutation in iUL49 and carrying the expression cassette of Flag-tagged hDUT-N, the nuclear isoform of human dUTPase, (F-hDUTn) inserted into the intergenic region between vdUTPase and UL51. A substitution in the iUL49 gene shown in line 3 was designed to have no effect on the amino acid sequence of VP22.

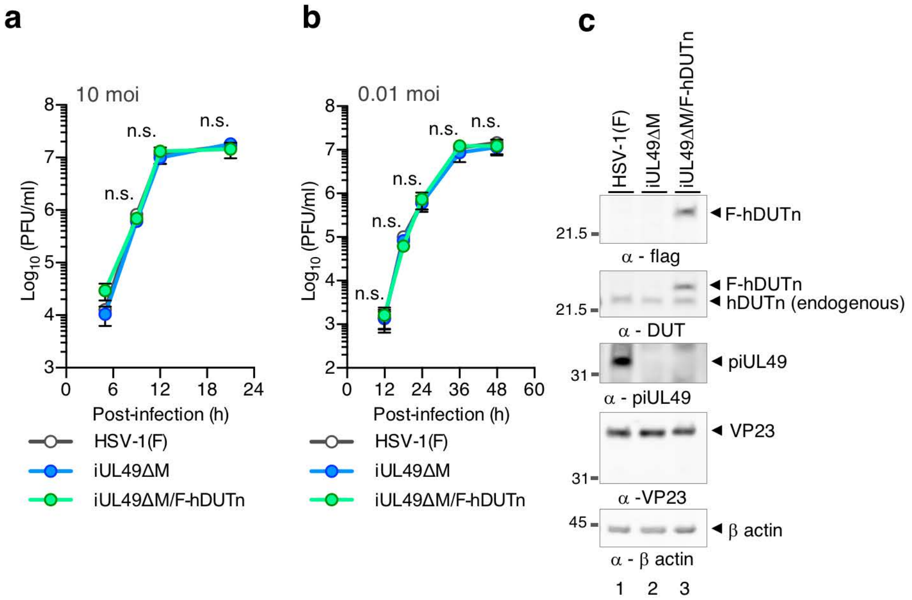


**Supplementary Fig. 34. Characterization of a recombinant virus iUL49DM/F-hDUTn. a, b,** Vero cells were infected with wild-type HSV-1(F), iUL49ΔM or iUL49ΔM/F-hDUTn at a MOI of 10 (**a**) or 0.01 (**b**). Viral titers in infected cells were assayed as described in **Supplementary Fig. 9**. Each value represents the mean ± SEM of three independent experiments and were analyzed by one-way ANOVA followed by Tukey's test. n.s., not significant. **c,** HEp-2 cells infected with wild-type HSV-1(F), iUL49ΔM or iUL49ΔM/F-hDUTn at an MOI 10 for 12 h were lysed and analyzed by immunoblotting with antibodies to Flag, hDUT, piUL49, VP23 or β actin. Digital images are representative results of three independent experiments. Source data are provided as a Source Data file.

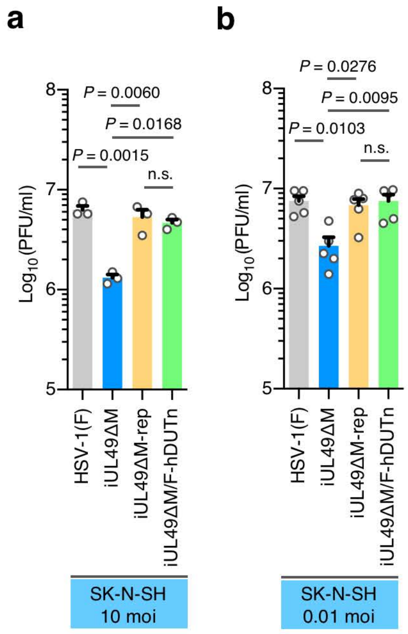


**Supplementary Figs. 35. Effect of piUL49 on HSV-1 replication in SK-N-SH cells. a, b,** SK-N-SH cells were infected with wild-type HSV-1(F), iUL49ΔM, iUL49ΔM-rep or iUL49ΔM/F-hDUTn at an MOI of 10 (**a**) or 0.01 (**b**). Total virus from the cell culture supernatants and infected cells was harvested at 24 (**a**) or 48 (**b**) h post-infection and viral titers in infected cells were assayed as described in **Supplementary Fig. 9**. Each value represents the mean ± SEM of three (**a**) or five (**b**) independent experiments. The indicated *P*-values were obtained using one-way ANOVA followed by Tukey's test. n.s., not significant. Source data are provided as a Source Data file.

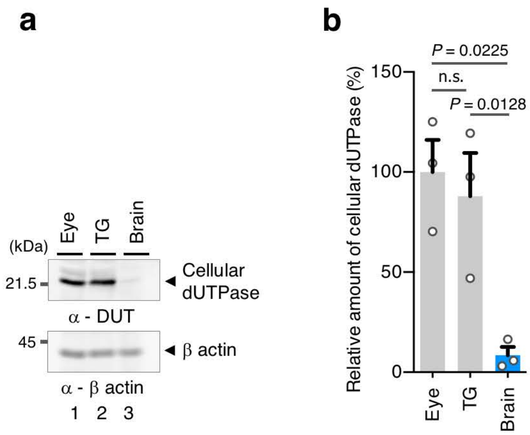


**Supplementary Fig. 36. Accumulation of cellular dUTPase in mouse eyes, TGs and brains. a,** The eyes, TGs and brains from eight 4-week-old female mice were lysed and analyzed by immunoblotting with antibodies to dUTPase (DUT) and β actin. Digital images are representative results of three independent experiments. **b,** The amounts of cellular dUTPase proteins in (**a**) were quantitated and normalized to those of β actin proteins. Each value is the mean ± SEM of the results of three independent experiments and is expressed relative to the mean for eyes, which was normalized to 100%. Each values were analyzed by one-way ANOVA followed by Tukey's test. n.s., not significant. Source data are provided as a Source Data file

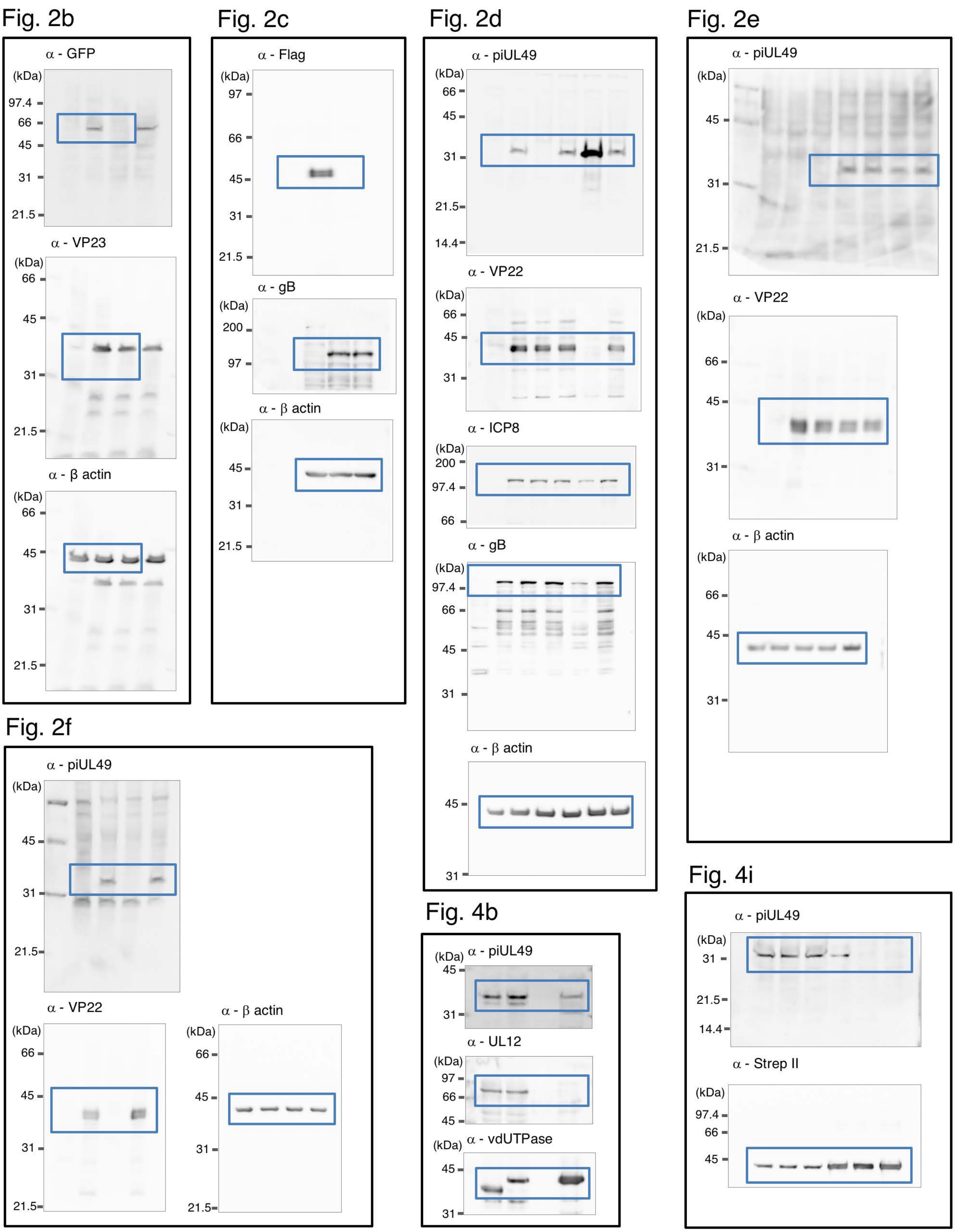


**Supplementary Fig. 37. Data sources of immunoblots-I.** Blue boxes indicate the cropped areas shown in the indicated figures. Molecular weight is indicated as a number.

Supplementary Fig. 4a

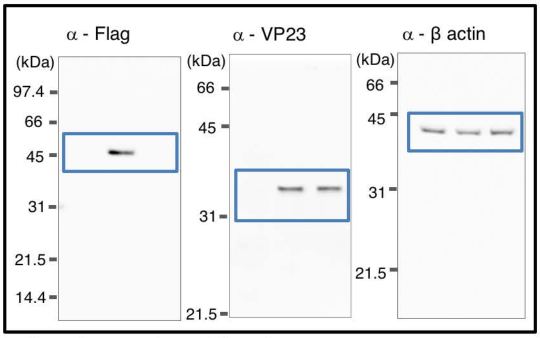


Supplementary Fig. 4b

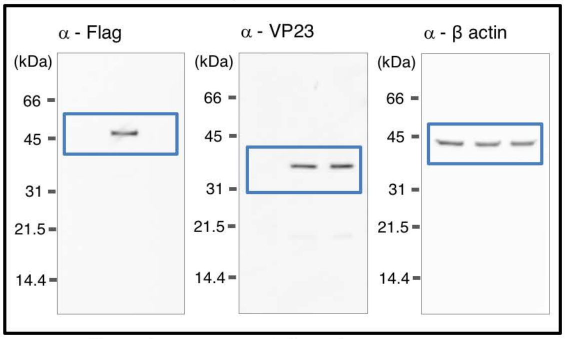


Supplementary Fig. 6

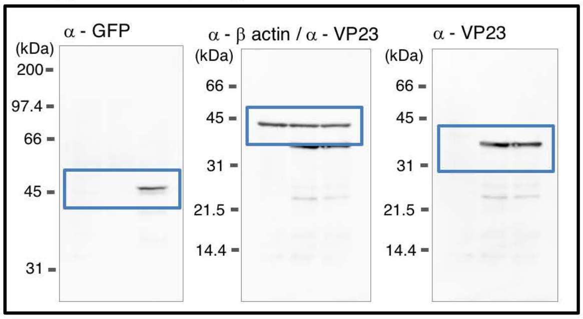


Supplementary Fig. 7b

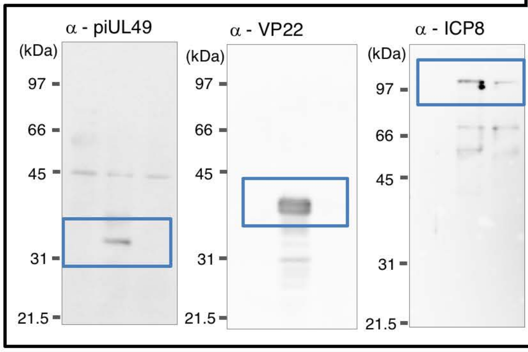


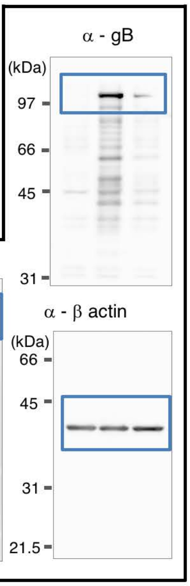


Supplementary Fig. 13a

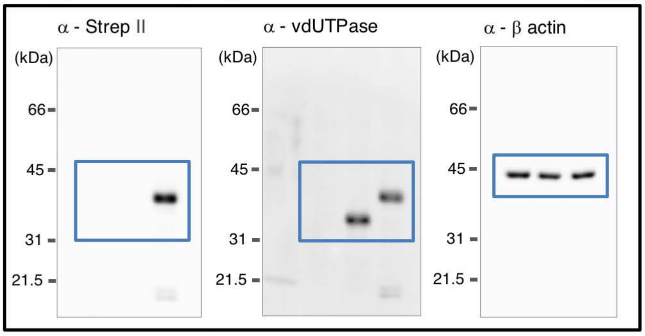


Supplementary Fig. 14

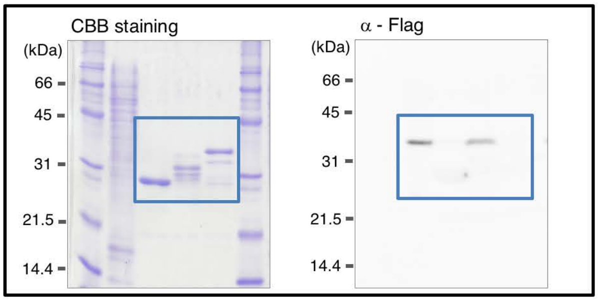


Supplementary Fig. 15a

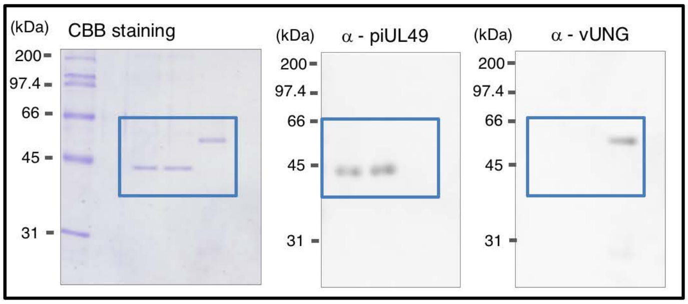


Supplementary Fig. 15b

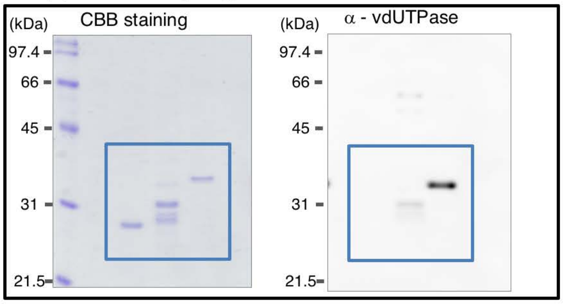


Supplementary Fig. 15c

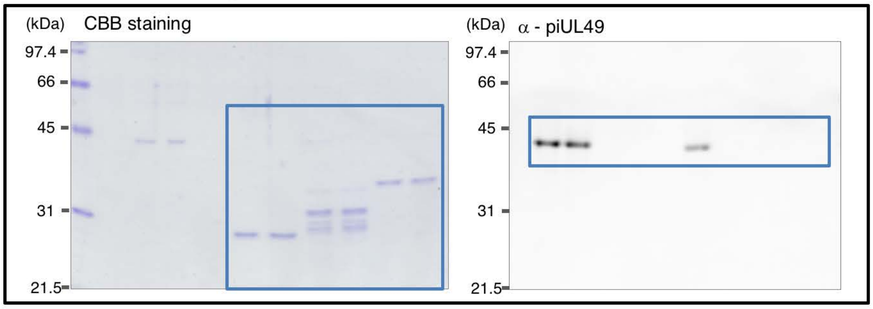


**Supplementary Fig. 38. Data sources of immunoblots and gels stained with CBB-I.** Blue boxes indicate the cropped areas shown in the indicated figures. Molecular weight is indicated as a number.

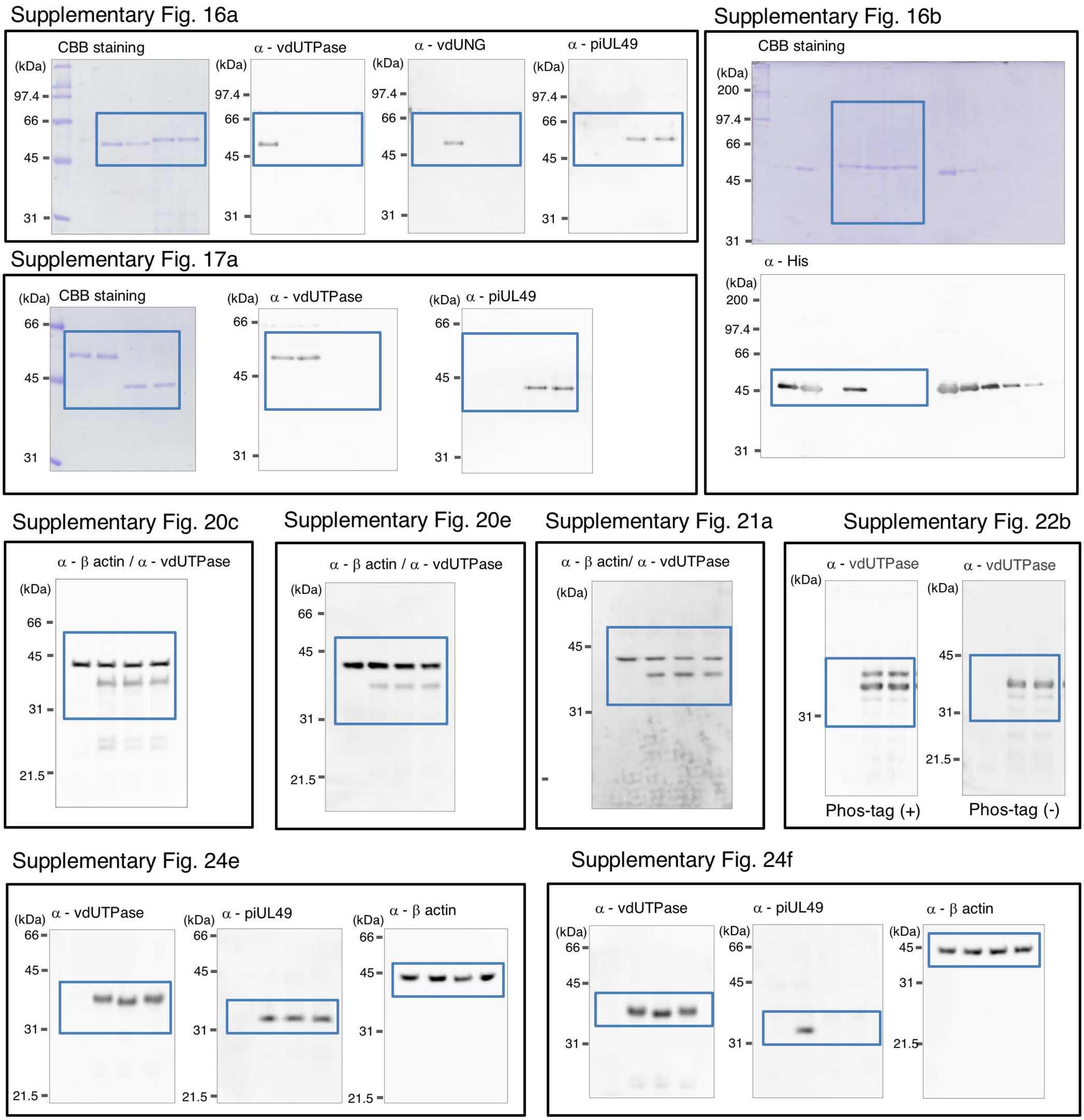


**Supplementary Fig. 39. Data sources of immunoblots and gels stained with CBB-II.** Blue boxes indicate the cropped areas shown in the indicated figures. Molecular weight is indicated as a number.

Supplementary Fig. 26a

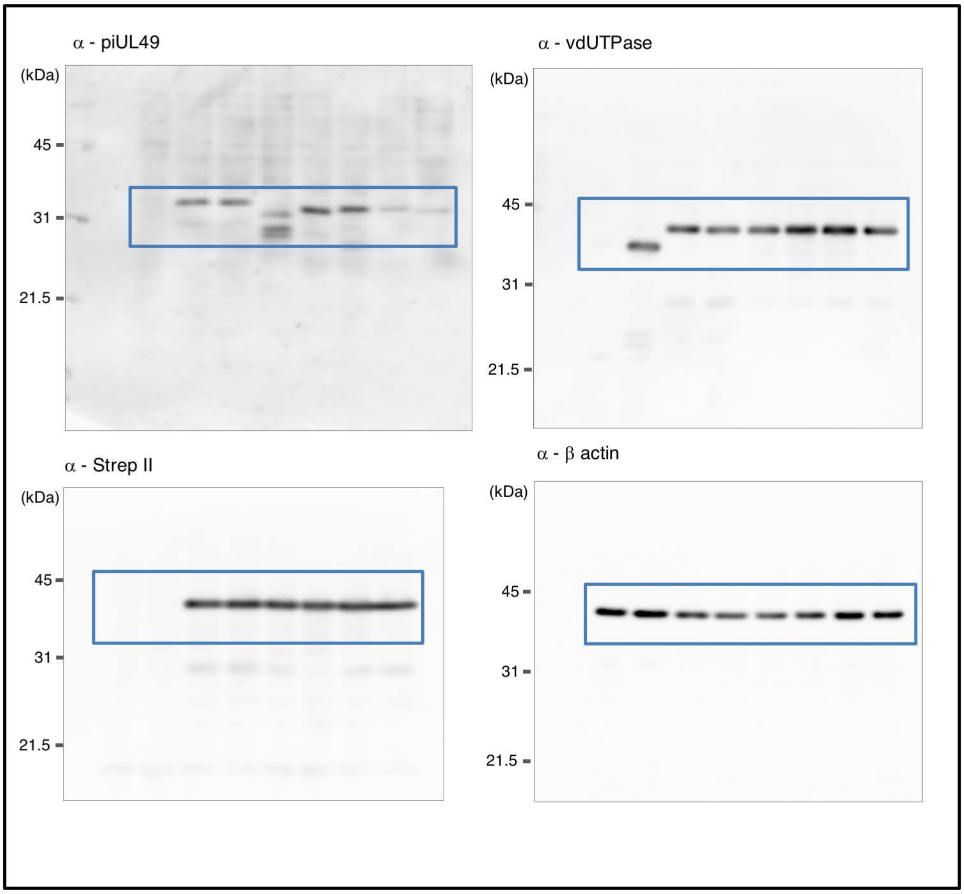


Supplementary Fig. 26b

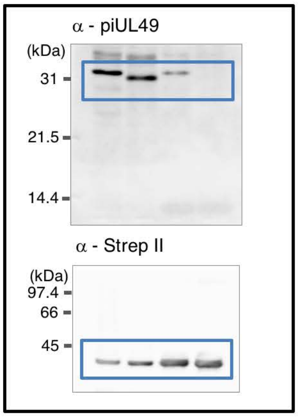


Supplementary Fig. 26c

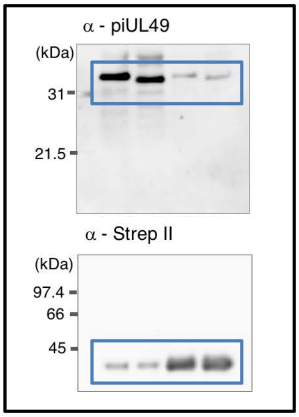


Supplementary Fig. 28a

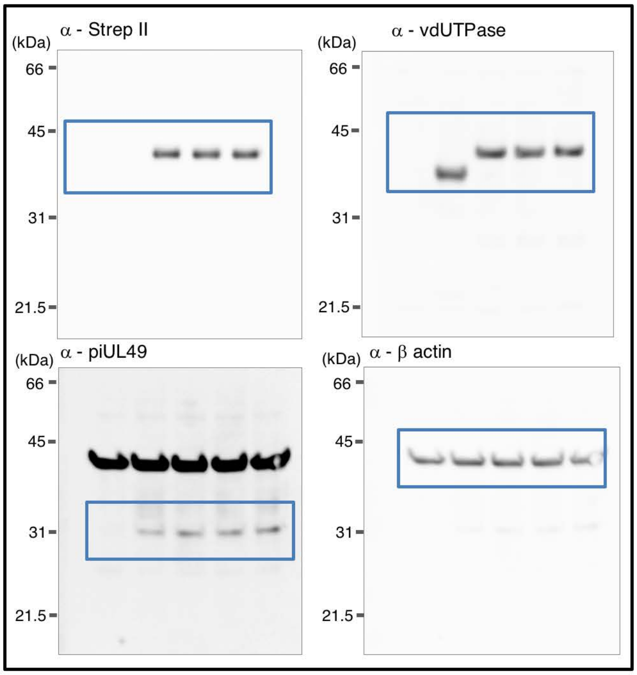


Supplementary Fig. 28b

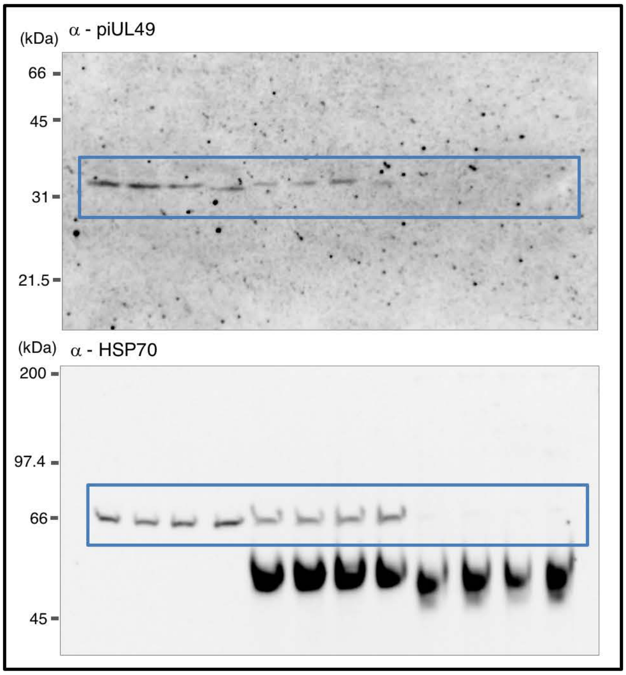


**Supplementary Fig. 40. Data sources of immunoblots-II.** Blue boxes indicate the cropped areas shown in the indicated figures. Molecular weight is indicated as a number.

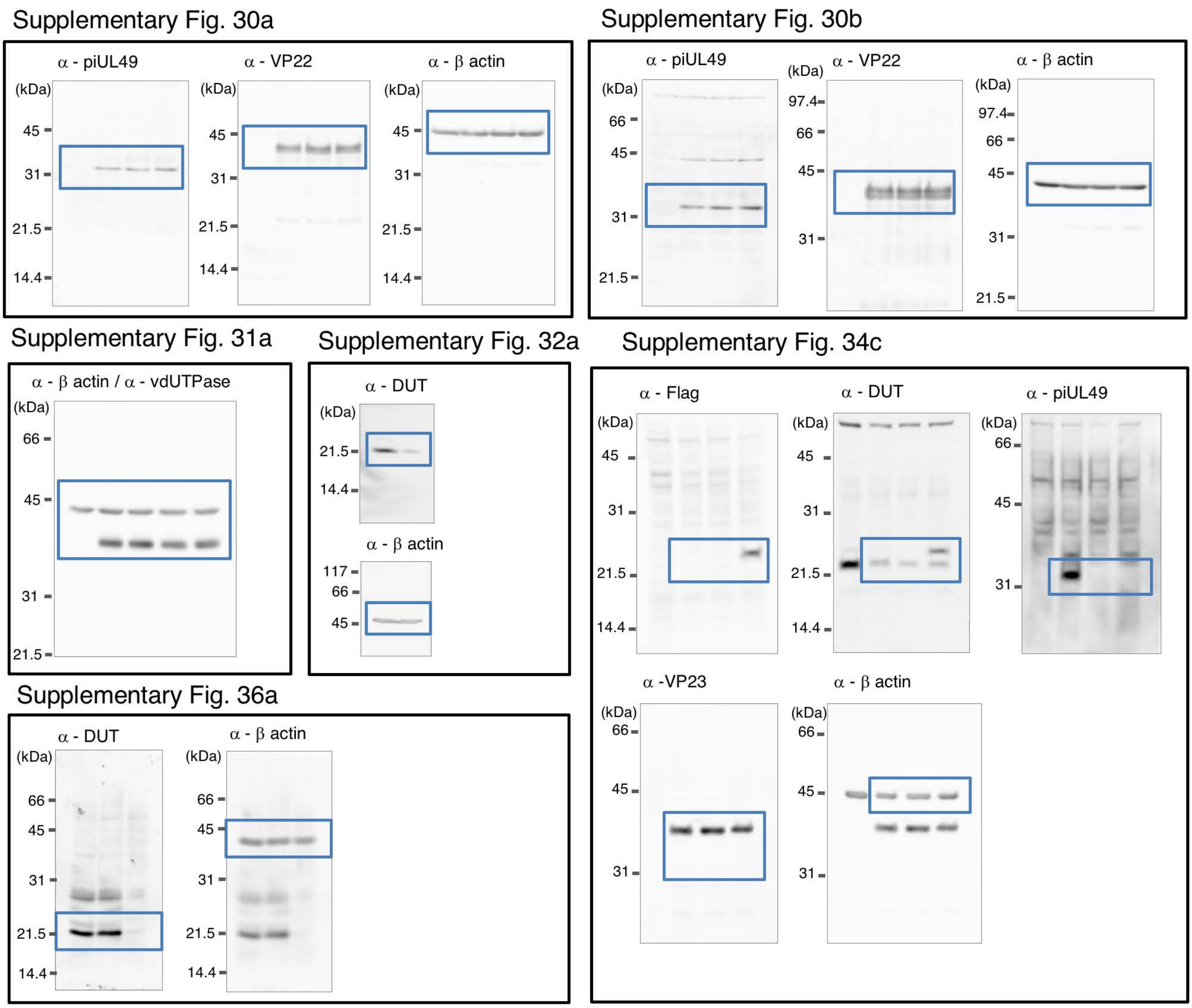


**Supplementary Fig. 41. Data sources of immunoblots-III.** Blue boxes indicate the cropped areas shown in the indicated figures. Molecular weight is indicated as a number.

**Supplementary Table 1. Summary of peptides using for the identification of previously cryptic orphan HSV-1 CDSs.**

| Gene name | Detected peptide | Highest confidence score | Mass deviation (ppm) | Number of PSMs | Modification | Charge |
|---|---|---|---|---|---|---|
| iICP0 | TPAVAGAPPLER | 99.000 | -0.644 | 5 | | 2 |
| | SRPTCAATPSR | 99.000 | -13.410 | 4 | Carbamidomethyl(C)@5 | 3 |
| iUL12 | LPAMGPAPAGPHDR | 99.000 | 2.340 | 3 | | 3 |
| | IHPPLGPPGSVFLAPVAEAQR | 99.000 | 1.343 | 15 | | 3 |
| | QPATGCTVFGGDVR | 99.000 | 1.440 | 5 | Carbamidomethyl(C)@6 | 2 |
| | QPATGCTVFGGDVR | 97.890 | 0.592 | 2 | Gln->pyro-Glu@N-term; Carbamidomethyl(C)@6 | 2 |
| | ADGPKPAVSLLR | 99.000 | -7.591 | 5 | | 3 |
| | LPGPSPQDPPGLLR | 99.000 | 0.215 | 14 | | 2 |
| | GLDGTPVPGGVPGIYPVDPEAQR | 99.000 | -0.788 | 10 | | 3 |
| iUL26a | RPCIAPHHTAPPTVLPR | 99.000 | -0.957 | 5 | Carbamidomethyl(C)@3 | 3 |
| | IAPHHTAPPTVLPR | 99.000 | 0.349 | 3 | | 2 |
| | IAPHHTAPPTVLPR | 99.000 | -1.836 | 10 | | 3 |
| | IAPHHTAPPTVLPR | 99.000 | -1.955 | 6 | Oxidation(P)@3 | 3 |
| | IAPHHTAPPTVLPR | 99.000 | -1.816 | 3 | Oxidation(H)@5 | 3 |
| | RPMPPLPR | 99.000 | -0.260 | 5 | | 2 |
| | MPQLPAR | 99.000 | 0.525 | 21 | | 2 |
| | SPPPPPDPNR | 99.000 | 0.006 | 9 | | 2 |
| | SPPPPPDPNRR | 99.000 | 0.278 | 11 | | 2 |
| | SPPPPPDPNRR | 99.000 | -1.018 | 10 | | 3 |
| | RPGPLSTPAAQHTW | 99.000 | 3.759 | 5 | | 3 |
| | RPGPLSTPAAQHTWTL | 99.000 | -0.573 | 2 | | 3 |
| | RPGPLSTPAAQHTWTLTR | 99.000 | -0.137 | 4 | | 3 |

| | | | | | | |
|---|---|---|---|---|---|---|
| | RPGPLSTPAAQHTWTLTR | 99.000 | 0.655 | 7 | | 4 |
| | STPAAQHTWTLTR | 99.000 | -1.533 | 3 | | 2 |
| | TLTRPAPPICSSLR | 99.000 | -5.534 | 4 | Carbamidomethyl(C)@10 | 3 |
| | TRPAPPICSSLR | 99.000 | 0.923 | 3 | Carbamidomethyl(C)@8 | 3 |
| | RPGPLSTPAAQHTWTLTRPAPPICSSLR | 99.000 | -0.125 | 3 | Carbamidomethyl(C)@24 | 4 |
| | RPGPLSTPAAQHTWTLTRPAPPICSSLR | 99.000 | -0.366 | 5 | Carbamidomethyl(C)@24 | 5 |
| | PAPPICSSLR | 99.000 | -0.126 | 4 | Carbamidomethyl(C)@6 | 2 |
| iUL26b | HRIPTGGIQR | 99.000 | 0.049 | 2 | Phospho(T)@5 | 3 |
| | IPTGGIQR | 99.000 | 0.091 | 6 | | 2 |
| iUL38 | YGPTSRPTTAGR | 99.000 | -0.465 | 2 | | 2 |
| | YGPTSRPTTAGR | 99.000 | 0.067 | 4 | | 3 |
| | SCGFSGGWCR | 99.000 | -0.445 | 6 | Carbamidomethyl(C)@2 and @9 | 2 |
| iUL44b | QRPQPNGPPR | 99.000 | -1.609 | 3 | Gln->pyro-Glu@N-term | 2 |
| | DPGNVLLGLGPDGQPAR | 99.000 | 0.102 | 10 | | 2 |
| | DPGNVLLGLGPDGQPAR | 99.000 | -0.260 | 7 | | 3 |
| | GDGGSAVQGDVHGR | 99.000 | -1.108 | 3 | | 2 |
| | GDGGSAVQGDVHGR | 99.000 | -0.606 | 10 | | 3 |
| iUL48 | VGQLLLGPVPVPAR | 99.000 | -0.510 | 3 | | 2 |
| | RADDAARPV | 99.000 | 0.613 | 3 | | 2 |
| iUL49 | LPPPAVAPYR | 96.740 | -0.894 | 14 | | 2 |
| | IMPSTGAR | 92.140 | 7.122 | 2 | | 2 |
| | RPLGPEGPDAHPPPPPGPPEPSGWR | 99.000 | -6.599 | 2 | | 4 |
| | RPPAAGNRPSQNPPHSQTPPR | 99.000 | -2.433 | 3 | | 4 |
| | RPPAAGNRPSQNPPHSQTPPR | 99.000 | -1.847 | 3 | | 5 |
| | RRPPAAGNRPSQNPPHSQTPPR | 99.000 | -5.900 | 2 | | 5 |
| | RGWPESCTLAPPPQTPTR | 99.000 | -1.927 | 6 | Carbamidomethyl(C)@7 | 3 |
| | HGPPGWPALTSASSAPR | 99.000 | -5.144 | 10 | | 3 |
| iUL50 | STPAASWSFLR | 99.000 | 0.000 | 5 | | 2 |

| | | | | | | |
|---|---|---|---|---|---|---|
| | LLFTTLR | 99.000 | -1.107 | 2 | | 2 |
| | QSAVSGPAGR | 99.000 | 5.106 | 5 | | 2 |
| | QCQPPAPPDTR | 99.000 | 0.722 | 6 | Carbamidomethyl(C)@2 | 2 |
| | QCQPPAPPDTR | 99.000 | 1.607 | 2 | Carbamidomethyl(C)@2 | 3 |
| | LLPDGILPSPSGPAHVVPF | 99.000 | 0.476 | 2 | | 2 |
| | LLPDGILPSPSGPAHVVPFAAGVR | 99.000 | -2.195 | 2 | | 3 |
| | NLGHAPAQHHTGGGGSIR | 99.000 | 0.752 | 2 | | 3 |
| | VQASLFGDQTVRPGV | 99.000 | -1.700 | 4 | | 2 |
| | VQASLFGDQTVRPGV | 99.000 | 3.377 | 5 | | 3 |
| iUs1 | QSAVSGPAGR | 99.000 | 5.106 | 5 | | 2 |
| | QCQPPAPPDTR | 99.000 | 0.722 | 6 | Carbamidomethyl(C)@2 | 2 |
| | QCQPPAPPDTR | 99.000 | 1.607 | 2 | Carbamidomethyl(C)@2 | 3 |
| | LLPDGILPSPSGPAHVVPF | 99.000 | 0.476 | 2 | | 2 |
| | LLPDGILPSPSGPAHVVPFAAGVR | 99.000 | -2.195 | 2 | | 3 |
| | NLGHAPAQHHTGGGGSIR | 99.000 | 0.752 | 2 | | 3 |
| | VQASLFGDQTVRPGV | 99.000 | -1.700 | 4 | | 2 |
| | VQASLFGDQTVRPGV | 99.000 | 3.377 | 5 | | 3 |

**Supplementary Table 2. Summary of identified HSV-1 CDSs by BONCAT-based MS-analyses.**

| Gene name | Amino acid sequence[a,b] | Transcript | CDS | Amino acid size | Conservation (%)[c] |
|---|---|---|---|---|---|
| iICP0 | *GGGGERGNSWALIDAGNPPILTRPPFFPLSPPRMSGCFPATETCRTAATLRRRPKWGGGGTP<br>TTMTTTPPPRRTARTRNCSRRGCWGRRAWMGGRSRGGAPPARKTPAVAGAPPLERTGGAT<br>RATCAPCARMRSRPTCAATPSRACTASASRA* | ☑ | 3057…<br>3419 | 120 | 100 |
| iUL12 | *LYVVGVGDPQRAALPYTSRDVRAPPARVAARRPRPSGHRSPLGPPGLSVFPGRGPRGGGYG<br>GPRTRPAPMAPDAPRPPGRRGRRGAPAPHGVLRGGHAKPGRLPAMGPAPAGPHDRIHPPL<br>GPPGSVFLAPVAEAQRQPATGCTVFGGDVRAGERADGPKPAVSLLRGPRGRRRRGRRRHAAL<br>YLPRTQRPRRRERAYVWGPHGRSHGDGRGVPGYSRLSSGHSRLPGPSPQDPPGLLRGQMPG<br>QVRFRPHGPQRPHGLRVRGLDGTPVPGGVPGIYPVDPEAQRAILRARARPRPGGGSRHARPG<br>LVRGPRLGRKKAVLRRGSGLGGVK* | ☑ | 26227…<br>25442 | 261 | 100 |
| iUL26a | *RLCSRNWRTCGLGPAPPMECTRRWRTIALRWGSRNQQRPTRPFVPRRPCIAPHHTAPPTVL<br>PRVRRPMPPLPRMPQLPARQARHRPHVLPPRRAPLYRRSPRSPPPPPDPNRRHPTRRPGPLST<br>PAAQHTWTLTRPAPPICSSLR* | ☑ | 52234…<br>52620 | 128 | 100 |
| iUL26b | *PVPNRPSARGRLAPGTAPHEPRSGIGHPGPRAARRRELPVDPGLPLQPARRRPRRAPTPAAF<br>RVWFPGCGGGRGLWASRRGSFPALPSPRRPSVSRGAVLGTQPTRGADSRVGGGHSRGPPGG<br>RSDGRGRPWGPGVGKASPVRGGAVGVLLRPGRTGRGLPVLPRGGSRRAARGRLSARGPPVS<br>RDQRDHHGADGGGDVSAAGTGAHAGSDQRPLWNVHAGGALSPSGGGAGTNNDPPGPLSP<br>GGRVSPPTTQRPLRSSPGSGVPCPHSPVCPSCLPARPATAPMSFHPDARPSTDGARVPPRRHR<br>IPTGGIQRGGRGPCQRQQRSTRGR* | ☑ | 52442…<br>52582 | 46 | 100 |

| | | | | | |
|---|---|---|---|---|---|
| iUL38 | *WRRPPWGRGEPRAGARARASCRLTSWWRRVPPRTTRATPPMRYGPTSRPTTAGRGWGRAWIVFPSVCAPWFTRTSSPTRSCGFSGGWCRGSPRTS* | ☑ | 85037…<br>85204 | 55 | 100 |
| iUL44b | *TPRARVVRPPRFIGPVRLAGADPMPVSEFHPHGVPPPDMALLHGSVPPNRSGSRPRGGPDEHHRPTRGTPGVRQRPQPNGPPRALGGGGRPGRRPSVVFCHRAAADPAADYRRGDARDPGNVLLGLGPDGQPARVRDVGARPHVPPPVSDPPAPRGDGGSAVQGDVHGRRLLPA* | ☑ | 96638…<br>97093 | 151 | 100 |
| iUL48 | *IPINAAQRCGGMGGRVRPRTRPNRHSRPRRRGLPDASGHPRRPRALLRSALSFLPRRATGAGGELSNRVGQLLLGPVPVPARQRPAAAPPGAHARTRSRPGRNAARHDHGQVLPRDRSSGACSVFAFVSIFDPRDPMGRVRRADDAARPV* | ☑ | 104632…<br>104213… | 139 | 100% |
| iUL49 | *SRVRGRFRAMSTRICTTPRLHVWRVPIVRLPPPAVAPYRHARARGARSVSSSTTSRIMPSTGARHPKTTNTRRSPGRGVPFPGRFCPARGLRGRLRRPLGPEGPDAHPPPPPGPPEPSGWRLRPPRPRRRRPPAAGNRPSQNPPHSQTPPRRRRQPDPRHPRRGWPESCTLAPPPQTPTRHGPPGWPALTSASSAPRSGAWRPCMPGWRRSSSGTCRVRAQTKTSTNSLASPPSA* | ☑ | 106238…<br>105558 | 226 | 100 |
| iUL50 | *RRFRRNTGTAYEKPSPSFQNARRMPVSTLSSVARSPSRQTAPRSCSHPSACSTRTPGPRPVMCWGGRRSTPAASWSFLRAGSPGTYVRLLFTTLRGFL* | ☑ | 107559…<br>107786 | 75 | 44.4 |
| iUs1 | *DGAGPVYPGNAAPGPHPVGPKPKCNATALGAPGPEAEQRTMDPRPGLHAPVYQSAVSGPAGRPGPPRQCQPPAPPDTRLLPDGILPSPSGPAHVVPFAAGVRRNLGHAPAQHHTGGGGSIRRHRGTRVQASLFGDQTVRPGV* | ☑ | 133064…13<br>3372 | 102 | 90 |

a; Putative CDSs are illustrated with black or red letters.

b; Peptide sequences detected by BONCAT-based MS-analyses are marked with both red and underlined letters.

c; Conservation among 80 HSV-1 strains is calculated by using bioinformatics analyses.

**Supplementary Table 3. Strains and accession numbers of HSV-1 using in bioinformatics analyses**

| Strains | Accession number | Strain names | Accession number |
|---|---|---|---|
| 0116209/India/2011 | KJ847330.1 [https://www.ncbi.nlm.nih.gov/nuccore/815859033] | 20-14-1 | KX265041.1 [https://www.ncbi.nlm.nih.gov/nuccore/KX265041.1] |
| 3M | KR011282.1 [https://www.ncbi.nlm.nih.gov/nuccore/KR011282.1] | 20-14-7 | KX265038.1 [https://www.ncbi.nlm.nih.gov/nuccore/KX265038.1] |
| 4M | KR011278.1 [https://www.ncbi.nlm.nih.gov/nuccore/KR011278.1] | 20-14-8 | KX265028.1 [https://www.ncbi.nlm.nih.gov/nuccore/KX265028.1] |
| 5-1-1 | KR011299.1 [https://www.ncbi.nlm.nih.gov/nuccore/KR011299.1] | 20-14-18 | KX265030.1 [https://www.ncbi.nlm.nih.gov/nuccore/KX265030.1] |
| 5-2-1 | KR011285.1 [https://www.ncbi.nlm.nih.gov/nuccore/KR011285.1] | 20-14-22 | KX265039.1 [https://www.ncbi.nlm.nih.gov/nuccore/KX265039.1] |
| 5-5-2 | KR011295.1 [https://www.ncbi.nlm.nih.gov/nuccore/KR011295.1] | 20-14-24 | KX265033.1 [https://www.ncbi.nlm.nih.gov/nuccore/KX265033.1] |
| 8S | KR011280.1 [https://www.ncbi.nlm.nih.gov/nuccore/KR011280.1] | 20L | KR011289.1 [https://www.ncbi.nlm.nih.gov/nuccore/KR011289.1] |
| 10-1-2 | KR011302.1 [https://www.ncbi.nlm.nih.gov/nuccore/KR011302.1] | 26S | KR011308.1 [https://www.ncbi.nlm.nih.gov/nuccore/KR011308.1] |
| 10-2-2 | KR011277.1 [https://www.ncbi.nlm.nih.gov/nuccore/KR011277.1] | 27S | KR011297.1 [https://www.ncbi.nlm.nih.gov/nuccore/KR011297.1] |
| 10-2-3 | KR011274.1 [https://www.ncbi.nlm.nih.gov/nuccore/KR011274.1] | 31XL | KR011304.1 [https://www.ncbi.nlm.nih.gov/nuccore/KR011304.1] |

| | | | |
|---|---|---|---|
| 10-5-1 | KR011301.1 [https://www.ncbi.nlm.nih.gov/nuccore/KR011301.1] | 34L | KR011275.1 [https://www.ncbi.nlm.nih.gov/nuccore/KR011275.1] |
| 10-6-1 | KR011296.1 [https://www.ncbi.nlm.nih.gov/nuccore/KR011296.1] | 36L | KR011279.1 [https://www.ncbi.nlm.nih.gov/nuccore/KR011279.1] |
| 10-6-2 | KR011306.1 [https://www.ncbi.nlm.nih.gov/nuccore/KR011306.1] | 47M | KR011305.1 [https://www.ncbi.nlm.nih.gov/nuccore/KR011305.1] |
| 10-6-3 | KR011284.1 [https://www.ncbi.nlm.nih.gov/nuccore/KR011284.1] | 57M | KR011276.1 [https://www.ncbi.nlm.nih.gov/nuccore/KR011276.1] |
| 11M | KR011294.1 [https://www.ncbi.nlm.nih.gov/nuccore/KR011294.1] | 66S | KR011281.1 [https://www.ncbi.nlm.nih.gov/nuccore/KR011281.1] |
| 12-12-2 | KR011298.1 [https://www.ncbi.nlm.nih.gov/nuccore/KR011298.1] | 76M | KR011300.1 [https://www.ncbi.nlm.nih.gov/nuccore/KR011300.1] |
| 12-12-67 | KR011286.1 [https://www.ncbi.nlm.nih.gov/nuccore/KR011286.1] | 82S | KR011307.1 [https://www.ncbi.nlm.nih.gov/nuccore/KR011307.1] |
| 16S | KR011303.1 [https://www.ncbi.nlm.nih.gov/nuccore/KR011303.1] | 83M | KR011310.1 [https://www.ncbi.nlm.nih.gov/nuccore/KR011310.1] |
| 19Lsyn | KR011293.1 [https://www.ncbi.nlm.nih.gov/nuccore/KR011293.1] | 914-A3 | KX265040.1 [https://www.ncbi.nlm.nih.gov/nuccore/KX265040.1] |
| 20-14-2 | KX265045.1 [https://www.ncbi.nlm.nih.gov/nuccore/KX265045.1] | 914-B | KX265043.1 [https://www.ncbi.nlm.nih.gov/nuccore/KX265043.1] |
| 914-B2 | KX265025.1 [https://www.ncbi.nlm.nih.gov/nuccore/KX265025.1] | E14 | HM585510.2 [https://www.ncbi.nlm.nih.gov/nuccore/HM585510.2] |
| 914-D2 | KX265026.1 | E15 | HM585503.2 |

| | | | |
|---|---|---|---|
| | [https://www.ncbi.nlm.nih.gov/nuccore/KX265026.1] | | [https://www.ncbi.nlm.nih.gov/nuccore/HM585503.2] |
| 914-E3 | KX265037.1 [https://www.ncbi.nlm.nih.gov/nuccore/KX265037.1] | E19 | HM585511.2 [https://www.ncbi.nlm.nih.gov/nuccore/HM585511.2] |
| 914-H2 | KX265031.1 [https://www.ncbi.nlm.nih.gov/nuccore/KX265031.1] | E25 | HM585506.2 [https://www.ncbi.nlm.nih.gov/nuccore/HM585506.2] |
| 914-N3 | KX265029.1 [https://www.ncbi.nlm.nih.gov/nuccore/KX265029.1] | E35 | HM585507.2 [https://www.ncbi.nlm.nih.gov/nuccore/HM585507.2] |
| 914-O2 | KX265022.1 [https://www.ncbi.nlm.nih.gov/nuccore/KX265022.1] | F | GU734771.1 [https://www.ncbi.nlm.nih.gov/nuccore/GU734771.1] |
| 914-Q | KX265024.1 [https://www.ncbi.nlm.nih.gov/nuccore/KX265024.1] | H166 | KM222726.1 [https://www.ncbi.nlm.nih.gov/nuccore/KM222726.1] |
| 914-R2 | KX265046.1 [https://www.ncbi.nlm.nih.gov/nuccore/KX265046.1] | H166syn | KM222727.1 [https://www.ncbi.nlm.nih.gov/nuccore/KM222727.1] |
| CJ311 | JN420338.1 [https://www.ncbi.nlm.nih.gov/nuccore/JN420338.1] | KOS | KM222722.1 [https://www.ncbi.nlm.nih.gov/nuccore/KM222722.1] |
| CJ970 | JN420341.1 [https://www.ncbi.nlm.nih.gov/nuccore/JN420341.1] | IV-2 | KX265023.1 [https://www.ncbi.nlm.nih.gov/nuccore/KX265023.1] |
| CJ994 | KR011283.1 [https://www.ncbi.nlm.nih.gov/nuccore/KR011283.1] | IV-6 | KX265032.1 [https://www.ncbi.nlm.nih.gov/nuccore/KX265032.1] |
| CR38 | HM585508.2 [https://www.ncbi.nlm.nih.gov/nuccore/HM585508.2] | IV-7 | KX265027.1 [https://www.ncbi.nlm.nih.gov/nuccore/KX265027.1] |
| 914-E3 | KX265037.1 [https://www.ncbi.nlm.nih.gov/nuccore/KX265037.1] | MacIntyre | KM222720.1 [https://www.ncbi.nlm.nih.gov/nuccore/KM222720.1] |

| | | | |
|---|---|---|---|
| E6 | HM585496.2 [https://www.ncbi.nlm.nih.gov/nuccore/HM585496.2] | R11 | HM585514.2 [https://www.ncbi.nlm.nih.gov/nuccore/HM585514.2] |
| E7 | HM585497.2 [https://www.ncbi.nlm.nih.gov/nuccore/HM585497.2] | R62 | HM585515.2 [https://www.ncbi.nlm.nih.gov/nuccore/HM585515.2] |
| E8 | HM585498.2 [https://www.ncbi.nlm.nih.gov/nuccore/HM585498.2] | RDH193 | KT425108.1 [https://www.ncbi.nlm.nih.gov/nuccore/KT425108.1] |
| E10 | HM585499.2 [https://www.ncbi.nlm.nih.gov/nuccore/HM585499.2] | S23 | HM585512.2 [https://www.ncbi.nlm.nih.gov/nuccore/HM585512.2] |
| E11 | HM585500.2 [https://www.ncbi.nlm.nih.gov/nuccore/HM585500.2] | S25 | HM585513.2 [https://www.ncbi.nlm.nih.gov/nuccore/HM585513.2] |
| E12 | HM585501.2 [https://www.ncbi.nlm.nih.gov/nuccore/HM585501.2] | SC16 | KX946970.1 [https://www.ncbi.nlm.nih.gov/nuccore/KX946970.1] |
| E13 | HM585502.2 [https://www.ncbi.nlm.nih.gov/nuccore/HM585502.2] | ZW6 | KX424525.1 [https://www.ncbi.nlm.nih.gov/nuccore/KX424525.1] |
| | | 17 | FJ593289.1 [https://www.ncbi.nlm.nih.gov/nuccore/FJ593289.1] |

**Supplementary Table 4. Host cell proteins that coimmunoprecipitated with FLAG-piUL49**

| Gene symbol | Ptotein name | Number of detected peptides |
|---|---|---|
| PARP1 | poly ADP-ribose polymerase 1 | 64 |
| RPL5 | 60S ribosomal protein L5 | 54 |
| KIAA1104\|PITRM1 | KIAA1104 family | 53 |
| PITRM1 | presequence protease; mitochondrial or mitochondrial (isoform unknown) | 50 |
| CLPX | ATP-dependent Clp protease ATP-binding subunit; clpX-like or clpX-like, (isoform unknown) | 49 |
| POLDIP2 | polymerase delta-interacting protein; 2 or 2 (isoform unknown) | 37 |
| NPM1 | nucleophosmin or nucleophosmin (isoform unknown) | 36 |
| TRIM26 | tripartite motif-containing protein; 26 or 26 (isoform unknown) | 30 |
| CHCHD2 | coiled-coil-helix-coiled-coil-helix domain-containing protein 2 | 23 |
| PDIA6 | protein disulfide-isomerase A6 | 20 |
| KPNA2 | importin subunit alpha-1 | 7 |
| HSPA1A\|HSPA1B\|HSPA6\|HSPA7 | heat shock; 70 kDa protein 1A, 1B, member 6, member 7 | 6 |
| DCAF7 | DDB1- and CUL4-associated factor 7 | 6 |
| B-ALPHA-1\|TUBA1A\|TUBA1B\|TUBA1C | tubulin alpha family | 5 |
| B-ALPHA-1\|TUBA1A\|TUBA1B\|TUBA1C\|TUBA3C\|TUBA3D\|TUBA3E | tubulin alpha family | 5 |
| YWHAE | 14-3-3 protein; epsilon or epsilon (isoform unknown) | 4 |

**Supplementary Table 5. Oligonucleotide sequences for the construction of plasmids and recombinant viruses**

| Plasmid or recombinant virus | Sequence (5'-3') |
|---|---|
| pBS-Venus-KanS | 5'-GCCTGCAGTGCTTCGCCCGCTACCCCGAGGATGACGACGATAAGTAGGG-3' |
| | 5'-GCCTGCAGGCCGTAGCCCAGGGTGGTCACAACCAATTAACCAATTCTGATTAG-3' |
| pMAL-iUL49-P3 | 5'-GCGAATTCCCGCGTCGACGGCGCCAACC-3' |
| | 5'-GCCTGCAGTCACGCGGATGGTGGTGATG-3' |
| pMAL-vUNG-P1 | 5'-GCGAATTCATGAAGCGGGCCTGCAGCCG-3' |
| | 5'-GCGTCGACCTAAGGCGACACCCCCCCGTTTG-3' |
| pGEX-vdUTPase-P1 | 5'-GCGAATTCCACGGGGACGGCGTACGAGA-3' |
| | 5'-GCGTCGACCTATACGTGCCCGGGGAGCCAGC-3' |
| pGEX-vdUTPase-P2 | 5'-GCGAATTCTGTGCGTTTGTTGTTTACAA-3' |
| | 5'-GCGTCGACCTAAATACCGGTAGAGCCAA-3' |
| pE-SUMO-vdUTPase or pE-SUMO-vdUTPaseD97A | 5'-GAACAGATTGGAGGTATGAGTCAGTGGGGATCCGG-3' |
| | 5'-ATTCGGATCCTCTAGCTAAATACCGGTAGAGCCAA-3' |
| pE-SUMO-vUNG | 5'-GAACAGATTGGAGGTATGAAGCGGGCCTGCAGCCG-3' |
| | 5'-ATTCGGATCCTCTAGTCAAACCGACCAGTCGATGG-3' |
| pEu-GST-iUL49 or pEu-GST-iUL49R83L | 5'-GCGATATCATGAGTACGAGGATCTGTAC-3' |
| | 5'-GCGGATCCTCACGCGGATGGTGGTGATG-3' |
| pFlag-iUL49 | 5'-GCAAGCTTATGAGTACGAGGATCTGTAC-3' |

| | |
|---|---|
| | 5’-GCGAATTCTCACGCGGATGGTGGTGATG-3’ |
| pcDNA-SE | 5’-AGCTTACCATGGCTAGCTGGAGCCACCCGCAGTTCGAGAAAGGTGGAGGTGCCCGAGGTGGATCGGGAGGTGGATCGTGGAGCCACCCGCAGTTCGAAAAAGGAGGTTCAGAGAATTTGTATTTTCAGGGTG-3’ |
| | 5’-AATTCACCCTGAAAATACAAATTCTCTGAACCTCCTTTTTCGAACTGCGGGTGGCTCCACGATCCACCTCCCGATCCACCTCGGGCACCTCCACCTTTCTCGAACTGCGGGTGGCTCCAGCTAGCCATGGTA-3’ |
| pcDNA-SE-iUL49 or pcDNA-SE-iUL49R83L | 5’-GCGAATTCATGAGTACGAGGATCTGTAC-3’ |
| | 5’-GCGATATCTCACGCGGATGGTGGTGATG-3’ |
| piUL49R9H3/L12-KanS-pst+, piUL49R6H1/L7-KanS-pst+, piUL49R7/L7-KanS-pst+, piUL49R5H1/L6-KanS-pst+ or piUL49R7H1/L8-KanS-pst+ | 5’-GCCTGCAGCGGTCTGCGAGGGCAAAAACAGGATGACGACGATAAGTAGGG-3’ |
| | 5’-GCCTGCAGTCACGCGGATGGTGGTGATGCAACCAATTAACCAATTCTGATTAG-3’ |
| pRB-EGRp-Flag-hDUTn-polyA-KanS | 5’-GCACTAGTGCCACCATGGACTACAAAGACGATGACGACAAGCCCTGCTCTGAAGAGACACCCGCCATTTCACCC-3’ |
| | 5’-GCGAATTCTTAATTCTTTCCAGTGGAAC-3’ |
| Venus-iUL49 | 5’-CCAACCCGATCCAAGACACCCGCGCAGGGGCTGGCCAGAAGTGAGCAAGGGCGAGGAGCT-3’ |
| | 5’-GGCGCGTCGGGGTTTGGGGGGGCGGTGCTAAAGTGCAGCTCTTGTACAGCTCGTCCATGC-3’ |
| MEF-iUL49 | 5’-CCAACCCGATCCAAGACACCCGCGCAGGGGCTGGCCAGAAATGGAGCAAAAGCTCATTTC-3’ |
| | 5’-GGCGCGTCGGGGTTTGGGGGGGCGGTGCTAAAGTGCAGCTATCTTTGTCATCGTCGTCCT-3’ |
| Venus-iUL44b | 5’-CCGCGACCCAGGGAATGTATTACTTGGCCTGGGGCCGGATGTGAGCAAGGGCGAGGAGCT-3’ |
| | 5’-CACCCACGTCCCGTACTCGTGCGGGCTGTCCATCCGGCCCCTTGTACAGCTCGTCCATGC-3’ |
| Venus-iUL49ΔM, | 5’-GCCATACATGAAGACGGGGTGTAGTACAGATCCTCGTACTCGTCGCGCGGAACCTCCCGCAGGATGACGAC |

| | |
|---|---|
| MEF-iUL49ΔM or iUL49ΔM | GATAAGTAGGG-3’ |
| | 5’-CGCCGCTCCGTGAAGTCGGGTCCGCGGGAGGTTCCGCGCGACGAGTACGAGGATCTGTACCAACCAATTAACCAATTCTGATTAG-3’ |
| iUL49ΔM-rep | 5’-GCCATACATGAAGACGGGGTGTAGTACAGATCCTCGTACTCATCGCGCGGAACCTCCCGCAGGATGACGACGATAAGTAGGG-3’ |
| | 5’-CGCCGCTCCGTGAAGTCGGGTCCGCGGGAGGTTCCGCGCGATGAGTACGAGGATCTGTACCAACCAATTAACCAATTCTGATTAG-3’ |
| VP22ΔM | 5’-GGAACCTCCCGCGGACCCGACTTCACGGAGCGGCGAGAGGTCTAGGTTCCACGAACACGCTAGGAGGATGACGACGATAAGTAGGG-3’ |
| | 5’-TAATTGTCCGCGCATCCGACCCTAGCGTGTTCGTGGAACCTAGACCTCTCGCCGCTCCGTCAACCAATTAACCAATTCTGATTAG-3’ |
| VP22ΔM-rep | 5’-GGAACCTCCCGCGGACCCGACTTCACGGAGCGGCGAGAGGTCATGGTTCCACGAACACGCTAGGAGGATGACGACGATAAGTAGGG-3’ |
| | 5’-TAATTGTCCGCGCATCCGACCCTAGCGTGTTCGTGGAACCATGACCTCTCGCCGCTCCGTCAACCAATTAACCAATTCTGATTAG-3’ |
| ΔVP22/ΔiUL49 | 5’-ACGCCGCAGAACGCCCTCGAGTCGCCGTGGCCGCGTCGACGGTTCCACGAACACGCTAGGAGGATGACGACGATAAGTAGGG-3’ |
| | 5’-TAATTGTCCGCGCATCCGACCCTAGCGTGTTCGTGGAACCGTCGACGCGGCCACGGCGAC CAACCAATTAACCAATTCTGATTAG-3’ |
| ΔVP22/ΔiUL49-rep | 5’-TCACTCGACGGGCCGTCTGG-3’ |
| | 5’-TAATTGTCCGCGCATCCGAC-3’ |
| SE-vdUTPase | 5’-GAGCGAGCGCGGGACCGGGGGTTTTGGCTCTACCGGTATTATGGCTAGCTGGAGCCACCC-3’ |
| | 5’-CGTTTTTTATTGCCCGGAACGAACCCCAAGCTATGGGCTAACCCTGAAAATACAAATTCT-3’ |

| | |
|---|---|
| ΔVP22/ΔiUL49/Zeo+/SE-vdUTPase or ΔVP22/ΔiUL49/Zeo+ | 5'-ACGCCGCAGAACGCCCTCGAGTCGCCGTGGCCGCGTCGACACGCGGCCTTTTTACGGTTC-3' |
| | 5'-TAATTGTCCGCGCATCCGACCCTAGCGTGTTCGTGGAACCATACCGCACAGATGCGTAAG-3' |
| iUL49R9H3/L12_SE-vdUTPase, iUL49R6H1/L7_SE-vdUTPase, iUL49R7/L7_SE-vdUTPase, iUL49R5H1/L6_SE-vdUTPase or iUL49R7H1/L8_SE-vdUTPase | 5'-GGCGCGACGCCGCAGAACGCCCTCGAGTCGCCGTGGCCGCGTCGACGTCCTGCACCACGTCTGGAT-3' |
| | 5'-TAATTGTCCGCGCATCCGACCCTAGCGTGTTCGTGGAACCATGACCTCTCGCCGCTCCGT-3' |
| iUL49R83L or iUL49R83L_SE-vdUTPase | 5'-CGGACGCGGCGTCCCGTTTCCGGGGCGGTTTTGTCCGGCCCGGGGCCTGCTCGGGCGCCTCCGCCGCCCGC AGGATGACGACGATAAGTAGGG-3' |
| | 5'-GGTGTGCGTCCGGCCCCTCCGGACCCAGCGGGCGGCGGAGGCGCCCGAGCAGGCCCCGGGCCGGACAAAA C CAACCAATTAACCAATTCTGATTAG-3' |
| iUL49R3/L3 or iUL49R3/L3_SE-vdUTPase | 5'-CGGACGCGGCGTCCCGTTTCCGGGGCGGTTTTGTCCGGCCCTGGGCCTGCTCGGGCTCCTCCGCCGCCCGCA GGATGACGACGATAAGTAGGG-3' |
| | 5'-GGTGTGCGTCCGGCCCCTCCGGACCCAGCGGGCGGCGGAGGAGCCCGAGCAGGCCCAGGGCCGGACAAAA CCAACCAATTAACCAATTCTGATTAG-3' |
| iUL49R83L-rep | 5'-CGGACGCGGCGTCCCGTTTCCGGGGCGGTTTTGTCCGGCCCGGGGCCTGCGCGGGCGCCTCCGCCGCCCGC AGGATGACGACGATAAGTAGGG-3' |
| | 5'-GGTGTGCGTCCGGCCCCTCCGGACCCAGCGGGCGGCGGAGGCGCCCGCGCAGGCCCCGGGCCGGACAAAA CCAACCAATTAACCAATTCTGATTAG-3' |
| iUL49R3/L3-rep | 5'-CGGACGCGGCGTCCCGTTTCCGGGGCGGTTTTGTCCGGCCCGGGGCCTGCGCGGGCGCCTCCGCCGCCCGC AGGATGACGACGATAAGTAGGG-3' |

| | |
|---|---|
| | 5’-GGTGTGCGTCCGGCCCCTCCGGACCCAGCGGGCGGCGGAGGCGCCCGCGCAGGCCCCGGGCCGGACAAAACCAACCAATTAACCAATTCTGATTAG-3’ |
| vdUTPaseD97A or iUL49ΔM/vdUTPaseD97A | 5’-GCTATCCAGCCCAGGGCACCACGTAATACTGGGTCTTATCGCTTCGGGGTACCGCGGAACAGGATGACGACGATAAGTAGGG -3’ |
| | 5’-GCGCTACGACCACGGCCATAACGGTTCCGCGGTACCCCGAAGCGATAAGACCCAGTATTACAACCAATTAACCAATTCTGATTAG -3’ |
| vdUTPaseDA-rep or iUL49ΔM/vdUTPaseDA-rep | 5’-GCTATCCAGCCCAGGGCACCACGTAATACTGGGTCTTATCGACTCGGGGTACCGCGGAACAGGATGACGACGATAAGTAGGG-3’ |
| | 5’-GCGCTACGACCACGGCCATAACGGTTCCGCGGTACCCCGAGTCGATAAGACCCAGTATTACAACCAATTAACCAATTCTGATTAG-3’ |
| iUL49ΔM/F-hDUTn | 5’-TATCTCATCTTTCCTGTGTGTAGTTGTTTCTGTTGGAGGCCTGTGGGTTAACATTGATTATTGACTAGTTATTAATAG-3’ |
| | 5’-TCATCCAACCCGTGTGTTCTGTGTTTGTGGGATGGAGGGGCGGGTGTGATCCATAGAGCCCACCGCATCC-3’ |

## Supplementary Table 6. Summary of synthesized plasmids

| Plasmid | Synthesized DNA sequences[a,b] | Amino acid sequences of mutant piUL49s[b] |
|---|---|---|
| pUC57-iUL49R9H3/L12-pst+ | atgacctctcgccgctccgtgaagtcgggtccgcgggaggttccgcgcgatgagtacgaggatctgtactacaccccttcttcttgtatggctagtcccgatagtcctcctgccacctcccgccgtggcgccctacagactcgcgctcgccctaggggcgaggtccgtttcgtccagtacgacgagtctgattatgccctctacgggggctcttcttccgaagacgacgaacaccctgaggtcccccggactcggcgtcccgtttccggggcggttttgtccggcccggggcctgcgcgggcgcctccgccgcccgctgggtccggaggggccggacgcacacccaccaccgcccccgggcccccgaacccagcgggtggcgactaaggcccccgcggccccggcggcggagaccacccgcggcaggaaatcggcccagccagaatccgccgcactcccagacgcccccgcgtcgacggcgccaacccgatccaagacacccgcgcaggggctggccagaaagctgcactttagcaccgccccccaaaccccgacgcgccatggacccccgggtggccggctttaacaagcgcgtcttctgcgccgcggtcgggcgcctggcggccatgcatgcccggatggcggcggtccagctctgggacatgtcgcgtccgcgcacagacgaagacctcaacgaactccttggcatcaccaccatccgcgtgactgcagcggtctgcgagggcaaaaacctgcttcagcgcgccaacgagttggtgaatccagacgtggtgcaggacgtcgac | MSTRICTTPLLLVWLVPIVLLPPPAVAPYRLALALGARSVSSSTTSLIMPSTGALLPKTTNTLRSPGLGVPFPGRFCPARGLRGRLRRPLGPEGPDAHPPPPPGPPEPSGWRLRPPRPRRRRPPAAGNRPSQNPPHSQTPPRRRRQPDPRHPRRGWPESCTLAPPPQTPTRHGPPGWPALTSASSAPRSGAWRPCMPGWRRSSSGTCRVRAQTKTSTNSLASPPSA |
| pUC57-iUL49R6H1/L7-pst+ | atgacctctcgccgctccgtgaagtcgggtccgcgggaggttccgcgcgatgagtacgaggatctgtactacaccccgtcttcatgtatggcgagtcccgatagtccgcctgccacctcccgccgtggcgccctacagacacgcgcgcgcccgaggggcgaggtccgtttcgtccagtacgacgagtcggattatgccctctacgggggctcgtcatccgaagacgacgaacacccggaggtcccccggacgcggcgtcccgtttccggggctgttttgtccggccctgggcctgctcgggctcctcctcctcccgctgggtccggaggggccggacgcactcccaccaccgcccccgggcccccgaacccagcgggtggcgactaaggcccccgcggccccggcggcggagaccacccgcggcaggaaatcggcccagccagaatccgccgcactcccagacgcccccgcgtcgacggcgccaacccgatccaagacacccgcgcaggggctggccagaaagctgcactttagcaccgccccccaaaccccgacgcgccatggacccccgggtggccggctttaacaagcgcgtcttctgcgccgcggtcgggcgcctggcggccatgcatgcccggatggcggcggtccagctctgggacatgtcgcgtccgcgcacagacgaagacctcaacgaactccttggcatcaccaccatccgcgtgactgcagcggtctgcgagggcaaaaacctgcttcagcgcgccaacgagttggtgaatccagacgtggtgcaggacgtcgac | MSTRICTTPRLHVWRVPIVRLPPPAVAPYRHARARGARSVSSSTTSRIMPSTGARHPKTTNTRRSPGRGVPFPGLFCPALGLLGLLLLPLGPEGPDALPPPPPGPPEPSGWRLRPPRPRRRRPPAAGNRPSQNPPHSQTPPRRRRQPDPRHPRRGWPESCTLAPPPQTPTRHGPPGWPALTSASSAPRSGAWRPCMPGWRRSSSGTCRVRAQTKTSTNSLASPPSA |

| | | |
|---|---|---|
| pUC57-iUL49R7/L7-pst+ | atgacctctcgccgctccgtgaagtcgggtccgcgggaggttccgcgcgatgagtacgaggatctgtactacaccccgtcttcatgtatggcgagtcccgata<br>gtccgcctgccacctcccgccgtggcgccctacagacacgcgcgcgcccgaggggcgaggtccgtttcgtccagtacgacgagtcggattatgccctctac<br>gggggctcgtcatccgaagacgacgaacacccggaggtcccccggacgcggcgtcccgtttccggggcggttttgtccggcccggggcctgcgcgggc<br>gcctccgccgcccgctgggtccggaggggccggacgcacacccaccaccgcccccgggcccccgaacccagcgggtggctactaaggcccccgct<br>gcccctgctgctgagaccacccgcggcaggaaatctgcccagccagaatccgccgctctcccagacgcccccgcgtcgacggcgccaacccgatccaag<br>acacccgcgcaggggctggccagaaagctgcactttagcaccgccccccaaaccccgacgcgccatggacccccgggtggccggctttaacaagcgc<br>gtcttctgcgccgcggtcgggcgcctggcggccatgcatgcccggatggcggcggtccagctctgggacatgtcgcgtccgcgcacagacgaagacctca<br>acgaactccttggcatcaccaccatccgcgtgactgcagcggtctgcgagggcaaaaacctgcttcagcgcgccaacgagttggtgaatccagacgtggtg<br>caggacgtcgac | MSTRICTTPRLHVWRVPIVRLPPPAVAPYRH<br>ARARGARSVSSSTTSRIMPSTGARHPKTTNT<br>RRSPGRGVPFPGRFCPALGLRGRLRRPLGPE<br>GPDAHPPPPPGPPEPSGWLLRPPLPLLLRPP<br>AAGNLPSQNPPLSQTPPRRRRQPDPRHPRR<br>GWPESCTLAPPPQTPTRHGPPGWPALTSAS<br>SAPRSGAWRPCMPGWRRSSSGTCRVRAQT<br>KTSTNSLASPPSA |
| pUC57-iUL49R5H1/L6-pst+ | atgacctctcgccgctccgtgaagtcgggtccgcgggaggttccgcgcgatgagtacgaggatctgtactacaccccgtcttcatgtatggcgagtcccgata<br>gtccgcctgccacctcccgccgtggcgccctacagacacgcgcgcgcccgaggggcgaggtccgtttcgtccagtacgacgagtcggattatgccctctac<br>gggggctcgtcatccgaagacgacgaacacccggaggtcccccggacgcggcgtcccgtttccggggcggttttgtccggcccggggcctgcgcgggc<br>gcctccgccgcccgctgggtccggaggggccggacgcacacccaccaccgcccccgggcccccgaacccagcgggtggcgactaaggcccccgc<br>ggccccggcggcggagaccacccgcggcaggaaatcggcccagccagaatccgccgcactcccagacgcccccgcttctactgctccaacccgatcca<br>agactcccgctcaggggctggccagaaagctgcactttagcaccgccccccaaaccccgacgcgccatggacccccgggtggccggctttaacaagc<br>gcgtcttctgcgccgcggtcgggcgcctggcggccatgcatgcccggatggcggcggtccagctctgggacatgtcgcgtccgcgcacagacgaagacct<br>caacgaactccttggcatcaccaccatccgcgtgactgcagcggtctgcgagggcaaaaacctgcttcagcgcgccaacgagttggtgaatccagacgtgg<br>tgcaggacgtcgac | MSTRICTTPRLHVWRVPIVRLPPPAVAPYRH<br>ARARGARSVSSSTTSRIMPSTGARHPKTTNT<br>RRSPGRGVPFPGRFCPALGLRGRLRRPLGPE<br>GPDAHPPPPPGPPEPSGWRLRPPRPRRRRP<br>PAAGNRPSQNPPRSQTPPLLLLQPDPRLPLR<br>GWPESCTLAPPPQTPTRHGPPGWPALTSAS<br>SAPRSGAWRPCMPGWRRSSSGTCRVRAQT<br>KTSTNSLASPPSA |
| pUC57-iUL49R7H1/L8-pst+ | atgacctctcgccgctccgtgaagtcgggtccgcgggaggttccgcgcgatgagtacgaggatctgtactacaccccgtcttcatgtatggcgagtcccgata<br>gtccgcctgccacctcccgccgtggcgccctacagacacgcgcgcgcccgaggggcgaggtccgtttcgtccagtacgacgagtcggattatgccctctac<br>gggggctcgtcatccgaagacgacgaacacccggaggtcccccggacgcggcgtcccgtttccggggcggttttgtccggcccggggcctgcgcgggc<br>gcctccgccgcccgctgggtccggaggggccggacgcacacccaccaccgcccccgggcccccgaacccagcgggtggcgactaaggcccccgc<br>ggccccggcggcggagaccacccgcggcaggaaatcggcccagccagaatccgccgcactcccagacgcccccgcgtcgacggcgccaacccgatc | MSTRICTTPRLHVWRVPIVRLPPPAVAPYRH<br>ARARGARSVSSSTTSRIMPSTGARHPKTTNT<br>RRSPGRGVPFPGRFCPARGLRGRLRRPLGPE<br>GPDAHPPPPPGPPEPSGWRLRPPRPRRRRP<br>PAAGNRPSQNPPHSQTPPRRRRQPDPRHP |

| | | |
|---|---|---|
| | caagacacccgcgcaggggctggccagaaagctgcactttagcaccgcccccccaaaccccgacgctccttggacccccccgggtggccggctttaacaa gcgcgtcttctgcgccgctgtcgggcgcctggctgccatgcatgcccggatggctgctgtccagctctgggacatgtctcgtcctcgcacagacgaagacct caacgaactccttggcatcaccaccatccgcgtga<u>ctgcag</u>cggtctgcgagggcaaaaacctgcttcagcgcgccaacgagttggtgaatccagacgtgg tgcaggacgtcgac | RRGWPESCTLAPPPQTPTLLGPPGWPALTS ASSAPLSGAWLPCMPGWLLSSSGTCLVLAQ TKTSTNSLASPPSA |

a; iUL49 or *Pst*1 sequences are illustrated with black or underlined letters, respectively.

b; Mutated sequences or amino acids are marked with red letters.
