# Supplementary material for: Identification of a herpes simplex virus 1 gene encoding neurovirulence factor by chemical proteomics
Source: Nat Commun. 2020 Sep 29;11:4894. doi: 10.1038/s41467-020-18718-9 (PMC7524712; doi:10.1038/s41467-020-18718-9)
Supplement: Supplementary file 3 — Reporting Summary [file 41467_2020_18718_MOESM3_ESM.pdf]

nature research

Corresponding author(s): Yasushi Kawaguchi

Last updated by author(s): Aug 31, 2020

Reporting Summary

Nature Research wishes to improve the reproducibility of the work that we publish. This form provides structure for consistency and transparency in reporting. For further information on Nature Research policies, see Authors & Referees and the Editorial Policy Checklist.

Please do not complete any field with "not applicable" or n/a. Refer to the help text for what text to use if an item is not relevant to your study.
For final submission: please carefully check your responses for accuracy; you will not be able to make changes later.

Statistics

For all statistical analyses, confirm that the following items are present in the figure legend, table legend, main text, or Methods section.

n/a	Confirmed	
☐	☒	The exact sample size (*n*) for each experimental group/condition, given as a discrete number and unit of measurement
☐	☒	A statement on whether measurements were taken from distinct samples or whether the same sample was measured repeatedly
☐	☒	The statistical test(s) used AND whether they are one- or two-sided<br>*Only common tests should be described solely by name; describe more complex techniques in the Methods section.*
☐	☒	A description of all covariates tested
☐	☒	A description of any assumptions or corrections, such as tests of normality and adjustment for multiple comparisons
☐	☒	A full description of the statistical parameters including central tendency (e.g. means) or other basic estimates (e.g. regression coefficient) AND variation (e.g. standard deviation) or associated estimates of uncertainty (e.g. confidence intervals)
☐	☒	For null hypothesis testing, the test statistic (e.g. *F*, *t*, *r*) with confidence intervals, effect sizes, degrees of freedom and *P* value noted<br>*Give P values as exact values whenever suitable.*
☒	☐	For Bayesian analysis, information on the choice of priors and Markov chain Monte Carlo settings
☒	☐	For hierarchical and complex designs, identification of the appropriate level for tests and full reporting of outcomes
☒	☐	Estimates of effect sizes (e.g. Cohen's *d*, Pearson's *r*), indicating how they were calculated

Our web collection on statistics for biologists contains articles on many of the points above.

Software and code

Policy information about availability of computer code

Data collection	MS analyses were performed by a mass spectrometer (Triple TOF 5600+; AB Sciex). Immunoblotting or Immunofluorescence images were visualized using a ImageQuant LAS 4000 system (GE Healthcare Life Sciences) or LSM800 microscope with ZEN2.3 (Zeiss), respectively. Tritium signals were calculated by using a Tri-Carb 2100TR Liquid Scintillation Analyzer (Perkin Elmer) or Liquid scintillation counter LSC-5100 (Aloka). ELIZA assays were carried out using EnSpire multimode plate reader (Perkin Elmer). Plaque sizes were determined by IX73 microscope equipped with a digital DP80 camera (Olympus).
Data analysis	Protein plot software (Sciex); ImageQuant TL7.0 analysis software (GE Healthcare Life Sciences); ZEN2.3 software (Carl Zeiss); Prism 6.0 software (GraphPad Software, Inc); cellSens software (Olympus).

For manuscripts utilizing custom algorithms or software that are central to the research but not yet described in published literature, software must be made available to editors/reviewers. We strongly encourage code deposition in a community repository (e.g. GitHub). See the Nature Research guidelines for submitting code & software for further information.

Data

Policy information about availability of data

All manuscripts must include a data availability statement. This statement should provide the following information, where applicable:

- Accession codes, unique identifiers, or web links for publicly available datasets
- A list of figures that have associated raw data
- A description of any restrictions on data availability

Reported in the Data availability section (page 41), supplementary table 3 and supplementary figures 37 to 41.

Field-specific reporting

Please select the one below that is the best fit for your research. If you are not sure, read the appropriate sections before making your selection.

☒ Life sciences ☐ Behavioural & social sciences ☐ Ecological, evolutionary & environmental sciences

Life sciences study design

All studies must disclose on these points even when the disclosure is negative.

Sample size	No sample-size calculation was performed.
Data exclusions	No data were excluded.
Replication	In vitro assays were performed in 3-6 independent experiments. Animal experiments were reproduced with multiple replicates of each experiment (Viral titers of infected mice tissues, n = 8-26; dUTPase activities of mice tissues, n = 4; LD50 values of infected mice, 6 mice per experimental group).
Randomization	Animals were randomly divided into experimental groups.
Blinding	Blinding was not performed and is not relevant in this study. Virtually all the data are quantitative.

Reporting for specific materials, systems and methods

We require information from authors about some types of materials, experimental systems and methods used in many studies. Here, indicate whether each material, system or method listed is relevant to your study. If you are not sure if a list item applies to your research, read the appropriate section before selecting a response.

Materials & experimental systems

n/a	Involved in the study	
☐	☒	Antibodies
☐	☒	Eukaryotic cell lines
☒	☐	Palaeontology
☐	☒	Animals and other organisms
☒	☐	Human research participants
☒	☐	Clinical data

Methods

n/a	Involved in the study	
☒	☐	ChIP-seq
☒	☐	Flow cytometry
☒	☐	MRI-based neuroimaging

Antibodies

Antibodies used	Reported in the Methods section (Antibodies; page 28 and 29).
Validation	Antibodies against host factors or epitope-tags were obtained commercially and were validated for the species and application as statemented on the manufacturer's website. Antibodies against viral factors, except ICP8, VP23 and UL42, were validated by using knockout viruses to confirm the specificity.

Eukaryotic cell lines

Policy information about cell lines

Cell line source(s)	Vero, HEp-2, SK-N-SH or rabbit skin cells were kindly provided by Dr Bernard Roizman at the University of Chicago (Chicago, USA). HeLa or J774.A1 cells were obtained from Dr Shinobu Kitazume at the RIKEN (Saitama, Japan) or Dr Yasunobu Yoshikai at the Kyushu University (Fukuoka, Japan), respectively. HaCaT, HFFF-2, HEK293FT or HEK293 cells were purchased from the Cell Lines Service (CLS), the European Collection of Authenticated Cell Cultures (ECACC), the Thermo Fisher Scientific or he American Type Culture Collection (ATCC), respectively.
Authentication	No
Mycoplasma contamination	All cell lines were not tested.
Commonly misidentified lines (See ICLAC register)	N/A

Animals and other organisms

Policy information about studies involving animals; ARRIVE guidelines recommended for reporting animal research

Laboratory animals	Reported in the Methods section (Animal studies; page 31).
Wild animals	The study did not involve wild animals
Field-collected samples	The study did not involve samples collected from the field
Ethics oversight	Reported in the Methods section (Animal studies; page 32).

Note that full information on the approval of the study protocol must also be provided in the manuscript.

This checklist template is licensed under a Creative Commons Attribution 4.0 International License, which permits use, sharing, adaptation, distribution and reproduction in any medium or format, as long as you give appropriate credit to the original author(s) and the source, provide a link to the Creative Commons license, and indicate if changes were made. The images or other third party material in this article are included in the article's Creative Commons license, unless indicated otherwise in a credit line to the material. If material is not included in the article's Creative Commons license and your intended use is not permitted by statutory regulation or exceeds the permitted use, you will need to obtain permission directly from the copyright holder. To view a copy of this license, visit http://creativecommons.org/licenses/by/4.0/
