# Supplementary material for: Identification of a herpes simplex virus 1 gene encoding neurovirulence factor by chemical proteomics
Source: Nat Commun. 2020 Sep 29;11:4894. doi: 10.1038/s41467-020-18718-9 (PMC7524712; doi:10.1038/s41467-020-18718-9)
Supplement: Supplementary file 4 — Description of Additional Supplementary Files [file 41467_2020_18718_MOESM4_ESM.pdf]

Description of Additional Supplementary Files

File Name: Supplementary Data 1
Description: Information on peptides detected by BONCAT-based MS-analyses.

File Name: Supplementary Software 1
Description: Code used to compare the identified HSV-1(F) CDSs with the corresponding CDSs of 80 HSV-1 strains.
